# Natural Antioxidants as Regulators of Circular RNA Expression and Function

**DOI:** 10.1002/wrna.70023

**Published:** 2025-08-12

**Authors:** Aseela Fathima, Shadiya Fawzu Ameer, Rabia Ilhem Kerzabi, Roberta Giordo, Gheyath K. Nasrallah, Hatem Zayed, Gianfranco Pintus

**Affiliations:** ^1^ Department of Biomedical Sciences College of Health Sciences, QU Health, Qatar University Doha Qatar; ^2^ Department of Biomedical Sciences University of Sassari Sassari Italy; ^3^ Department of Medical Laboratory Sciences College of Health Sciences, Sharjah Institute for Medical Research, University of Sharjah Sharjah UAE

**Keywords:** circular RNAs (circRNAs), natural antioxidants, noncoding RNA therapeutics, oxidative stress–related diseases, redox regulationantioxidant–circRNA axis

## Abstract

Circular RNAs (circRNAs) are a class of noncoding RNAs characterized by covalently closed loop structures that confer high stability and diverse regulatory functions. Emerging evidence suggests that circRNAs modulate gene expression by acting as miRNA sponges, interacting with RNA‐binding proteins (RBPs), influencing transcription, and serving as translational templates. Their dysregulation has been linked to various diseases, including cancer, cardiovascular, neurodegenerative, and metabolic disorders. Oxidative stress, a common hallmark in these pathologies, can alter circRNA expression and function. Natural antioxidants, derived from dietary sources such as fruits, vegetables, herbs, and medicinal plants, offer a promising approach for restoring redox homeostasis and influencing the regulation of circRNA networks. This review provides a comprehensive overview of how different classes of natural antioxidants, including flavonoids, polyphenols, carotenoids, terpenoids, vitamins, and alkaloids, modulate circRNA expression and function in various disease contexts. Representative compounds such as quercetin, curcumin, resveratrol, astaxanthin, kaempferol, and genistein exhibit circRNA‐mediated actions that impact oxidative stress, inflammation, cell proliferation, apoptosis, and differentiation. The molecular mechanisms involve circRNA–miRNA–mRNA axes, interactions with RBPs, and modulation of epigenetic regulators and signaling pathways. We also discuss key challenges, including limited mechanistic understanding, bioavailability constraints, and the need for in vivo validation. Future perspectives emphasize the integration of antioxidant therapy with RNA‐targeted approaches, advanced delivery systems, and personalized profiling of circRNA. Collectively, the regulatory interplay between natural antioxidants and circRNAs represents a promising frontier in redox biology and RNA‐based therapeutics.

This article is categorized under:

RNA Interactions with Proteins and Other Molecules > Small Molecule‐RNA Interactions
RNA in Disease and Development > RNA in Disease

## Graphical abstract

Natural antioxidants found in food can modulate circular RNA activity, offering a novel strategy to counteract oxidative stress and its related diseases, including cardiovascular disorders, diabetes, and cancer. This emerging connection between diet and gene regulation holds promising potential for both therapy and prevention.

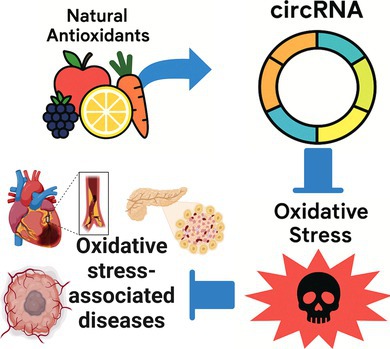

## 1 Introduction

**FIGURE 1:**
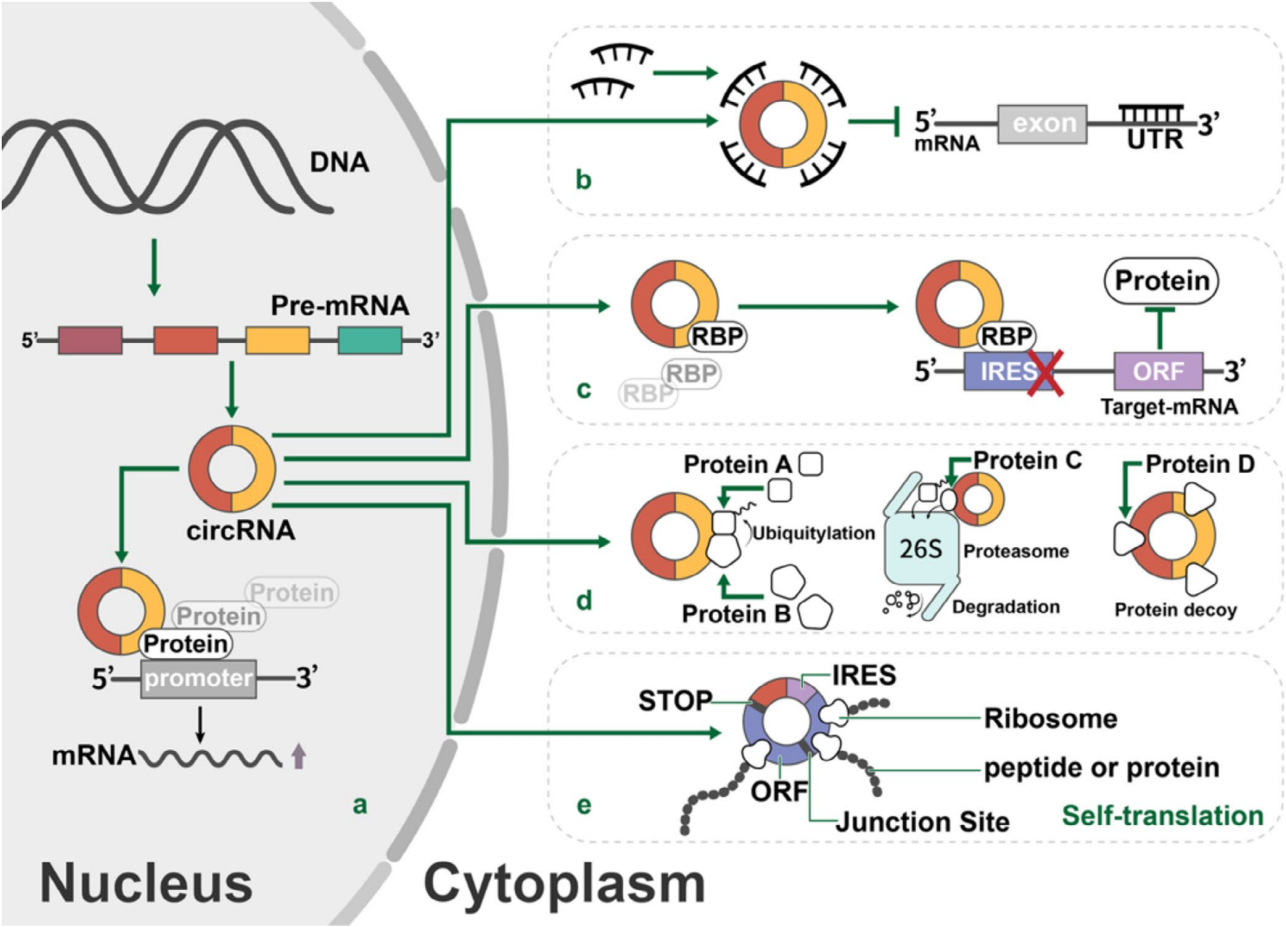
Schematic of circRNA Biogenesis and Functions. This schematic illustrates the generation of circRNAs and their diverse functions. (a) Back‐splicing of a pre‐mRNA leads to the formation of a covalently closed circRNA (exonic circRNA is shown); intron lariats escaping debranching form intronic circRNAs. (b) circRNAs can act as miRNA sponges, sequestering microRNAs and preventing them from repressing their targets. (c) circRNAs bind and sequester RNA‐binding proteins (RBPs), modulating RBP availability and function. (d) Certain circRNAs interact with transcriptional machinery (especially exon‐intron circRNAs in the nucleus) to regulate parent gene expression. (e) Some circRNAs contain start codons/IRES and encode peptides. Collectively, circRNAs influence gene expression at multiple levels, thereby contributing to the development and progression of disease.

Circular RNAs (circRNAs) are a distinct class of endogenous noncoding RNAs (ncRNAs) defined by a covalently closed loop structure lacking 5′ and 3′ ends, which renders them resistant to exonucleolytic degradation (e.g., by RNase R) and highly stable in cells (Jiang et al. 2021; Zheng et al. 2021). Initially discovered in viruses and viroids (Jiang et al. 2021), circRNAs were later identified in eukaryotic cells (Pisignano et al. 2023). They are primarily generated via back‐splicing, wherein a downstream 5′ splice donor is joined to an upstream 3′ splice acceptor, producing transcripts composed of exons (ecircRNAs), introns (ciRNAs), or both (EIciRNAs) (Figure 1) (Akhter 2018; Chen and Lu 2021). Their biogenesis is regulated by cis‐elements (e.g., inverted repeats) and trans‐acting factors such as RNA‐binding proteins (RBPs). For example, Quaking (QKI) promotes the formation of circRNA during the epithelial‐mesenchymal transition (EMT), whereas ADAR enzymes can inhibit its production by editing double‐stranded RNA structures (Conn et al. 2015; Pisignano et al. 2023).

Thousands of circRNAs have been identified across various tissues and species, often displaying cell‐type, developmental, or disease‐specific expression profiles (Chaudhary et al. 2020). Functionally, circRNAs serve as microRNA (miRNA) sponges and RBP decoys, modulating the activity of these regulatory molecules (Ikeda et al. 2023). Nuclear circRNAs (notably EIciRNAs) can influence the transcriptional activity of host genes, whereas others encode functional peptides (Babin et al. 2021). Dysregulated circRNA expression has been implicated in diverse diseases, including cancer, cardiovascular, and neurological disorders, functioning either as oncogenes or tumor suppressors (Babin et al. 2021).

Oxidative stress has emerged as a key driver of circRNA dysregulation (Giordo et al. 2021, 2024; Posadino et al. 2022; Yaacoub et al. 2024). Reactive oxygen and nitrogen species (ROS/RNS), such as superoxide and hydroxyl radicals, damage lipids, proteins, and nucleic acids, disturbing cellular homeostasis and contributing to disease progression (Boin et al. 2014; Fois et al. 2018; Giordo et al. 2022; Kattoor et al. 2017; Vono et al. 2016; Zinellu et al. 2016). These species can alter the expression and function of circRNAs through direct molecular interactions or activation of redox‐sensitive signaling pathways (Giordo et al. 2021, 2024; Posadino et al. 2022; Yaacoub et al. 2024).

Natural antioxidants—present in fruits, vegetables, herbs, and medicinal plants—counteract oxidative stress via reactive oxygen species (ROS) scavenging, metal chelation, and modulation of redox‐related ncRNA expression (Ramli, Cheriet, Posadino, et al. 2023; Ramli, Posadino, Giordo, et al. 2023; Ramli, Posadino, Zerizer, et al. 2023; Posadino et al. 2025, 2012). They may indirectly restore redox balance or directly modulate circRNA biogenesis and function by influencing regulatory pathways (Giordo et al. 2022; Ramli, Cheriet, Posadino, et al. 2023; Ramli, Posadino, Giordo, et al. 2023). In chronic oxidative stress, exogenous antioxidants, including flavonoids, polyphenols, carotenoids, vitamins, terpenoids, and alkaloids, may help reestablish redox homeostasis and preserve circRNA‐mediated regulatory networks (Ramli, Cheriet, Posadino, et al. 2023; Ramli, Posadino, Giordo, et al. 2023; Posadino et al. 2025; Hossain et al. 2022).

Mounting evidence suggests that natural antioxidants modulate circRNA expression and activity both indirectly (by attenuating oxidative stress) and directly (through redox‐sensitive pathways). Elucidating how specific antioxidant classes influence circRNA regulatory circuits is critical for identifying novel gene regulatory mechanisms and therapeutic targets. The following sections review these findings, organized by antioxidant class, with an emphasis on molecular mechanisms and outcomes related to circRNA. A summary of circRNA targets and their modulation by representative antioxidant compounds is provided in Table 1.

**TABLE 1:** Classes of natural antioxidants and representative circRNA targets.

Antioxidant class	Representative circRNA target(s)	Example compound(s) and context
Flavonoids (polyphenolic phytochemicals)	circHIAT1—acts as a sponge for miR‐19a‐3p, tumor‐suppressive when upregulated	*Quercetin* and *Baicalein* upregulate circHIAT1 in cancer models, inhibiting oncogenic miR‐19a and AKT/mTOR signaling (Hu et al. 2021; Li et al. 2024)
Carotenoids (lipid‐soluble terpenoids)	circTTP2—promotes cholesterol efflux via miR‐3073b‐5p sponging	*Astaxanthin* increases circTTP2 in macrophage foam cells, enhancing ABCA1‐mediated cholesterol efflux and reducing atherosclerosis (Zhang, Qiu, et al. 2023)
Polyphenols (non‐flavonoid)	circFOXP1—suppresses ferroptosis by sponging miR‐520a‐5p (upregulates SLC7A11)	*Curcumin* downregulates circFOXP1 in lung cancer cells, freeing miR‐520a‐5p to inhibit SLC7A11 and sensitizing cells to ferroptosis (Zhang, Yu, et al. 2024)
Vitamins (dietary antioxidants)	circHIPK2—a circRNA regulating differentiation via miR‐124‐3p (targeting MAPK1)	*All‐trans retinoic acid (Vitamin A)* treatment upregulates circHIPK2 during acute promyelocytic leukemia cell differentiation, enhancing miR‐124 activity and MAPK1 downregulation (Li, Ma, et al. 2018)
Terpenoids (isoprenoids, e.g., terpenes)	(No specific circRNA target has been identified to date)—indirect modulation of circRNA networks	*Ginsenoside Rb1* activates antioxidant pathways (e.g., Nrf2) that likely stabilize or normalize circRNA expression, though specific circRNAs remain unreported (Padmavathi and Ramkumar 2021).
Alkaloids (nitrogenous phytochemicals)	(No specific circRNA target has been identified to date)—indirect modulation of circRNA networks	*Berberine* and related alkaloids influence AMPK, JNK, and other stress‐response pathways, intersecting with circRNA regulatory hubs. Specific circRNAs have not yet been conclusively documented (Lu et al. 2015)

*Note:* Summary of major antioxidant classes (with example compounds) and circRNA molecules modulated by those compounds. Representative circRNA “targets” refer to circRNAs whose expression is altered (up‐ or down‐regulated) by antioxidants, affecting downstream pathways.

## 2 circRNA Biogenesis and Function

### 2.1 Biogenesis of circRNAs

circRNAs are produced through non‐canonical splicing mechanisms, including back‐splicing, intron pairing, and RBP‐mediated circularization. Their closed‐loop structure, lacking 5′ caps and 3′ poly(A) tails, confers exceptional stability. circRNAs may derive from exons, introns, UTRs, or intergenic regions, often involving exon skipping or intron retention. Exon skipping predominantly yields exonic circular RNAs (ecircRNAs), which comprise ~80% of identified circRNAs (Barbagallo et al. 2018; Pisignano et al. 2023). Intron pairing—facilitated by ALU repeats—produces stable ciRNAs with 2′–5′ phosphodiester bonds, whereas incomplete intron excision results in EIciRNAs (Zhen et al. 2022).

RBPs, such as muscleblind‐like proteins (MBNL), Quaking (QKI), and heterogeneous nuclear ribonucleoprotein L (HNRNPL), regulate circRNA biogenesis by either enhancing or inhibiting the circularization process. QKI promotes circRNA formation during EMT, whereas MBL binding inhibits linear splicing (Conn et al. 2015). Additionally, NF90/NF110 stabilize intronic RNA pairs during viral infection, though their cytoplasmic translocation reduces circRNA levels. ADAR enzymes modulate circularization by editing double‐stranded RNA regions (Pisignano et al. 2023). Thus, circRNA biogenesis is orchestrated by both cis‐elements and trans‐acting factors within a complex regulatory framework (Chen and Lu 2021).

### 2.2 Functions of circRNAs

circRNAs exert diverse functions depending on subcellular localization. In the nucleus, they regulate transcription, while in the cytoplasm, they act as miRNA sponges, protein decoys, scaffolds, and templates for translation (Geng et al. 2020; Greene et al. 2017).

As miRNA sponges, circRNAs sequester miRNAs to prevent their interaction with target mRNAs, enhancing gene expression through the competing endogenous RNA (ceRNA) mechanism. For example, ciRS‐7 binds to miR‐7 to regulate its targets (Wang et al. 2023; Zhao et al. 2020), and CDR1as, which contains 63 miR‐7 binding sites, upregulates miR‐7 target genes involved in tumor progression (Wang et al. 2023; Zhao et al. 2020). CircNRIP1 sponges miR‐149‐5p to modulate AKT1 expression and suppress gastric cancer cell proliferation (Zhang et al. 2019), while circ_0001667 sponges miR‐4458 to upregulate NCOA3, reversing Adriamycin resistance in MCF‐7 and DA‐MB 231 cells (Y. Cui et al. 2022). CircNR3C2 sponges miR‐513a‐3p to promote HRD1 expression and inhibit EMT in breast cancer (Fan et al. 2021), and ciRS‐7 also inhibits UBE2A in Alzheimer's disease via miR‐7 interaction (Akhter 2018).

circRNAs also bind to RBPs to modulate post‐transcriptional regulation, splicing, transport, and translation, forming stable RNA‐protein complexes (RPCs) with factors such as Argonaute (AGO), QKI, RNA Polymerase II (Pol II), Eukaryotic Initiation Factor 4A3 (EIF4A3), Adenosine Deaminase Acting on RNA (ADAR), and DEAH‐box Helicase 9 (DHX9) (Liu et al. 2019). These interactions govern cellular functions, including cell cycle and autophagy. For instance, circ‐Foxo3 binds to CDK2, arresting the cell cycle at the G1/S phase (Du et al. 2016), and CDR1as stabilizes mRNA transcripts through duplex formation (Hansen et al. 2011). TP53, acting as an RBP, directly interacts with circRNAs, contributing to tumor suppression (S. Chen et al. 2021). Circ‐Dnmt1 binds p53 and AUF1 to promote nuclear translocation and autophagy (Du et al. 2018), whereas circ‐Ccnb1 interacts with MDM2 and RBPs to enhance cell survival and proliferation (Chaudhary et al. 2020; Fang et al. 2018).

Despite lacking 5′ caps and 3′ tails, circRNAs can serve as translational templates through cap‐independent mechanisms, including internal ribosome entry sites (IRES) and m6A‐driven initiation (Tao et al. 2021). For instance, m6A enhances the IRES‐mediated translation of circZNF609. circRNAs with infinite open reading frames (ORFs) may undergo rolling‐circle translation independently of IRES, potentially yielding higher protein output than linear RNAs (Liu, Guo, et al. 2023). Although circRNA‐derived peptides often resemble truncated canonical proteins, some, such as circFNDC3B‐218aa, exhibit distinct functions, thereby expanding the proteomic landscape. Figure 1 summarizes the diverse molecular mechanisms by which circRNAs regulate gene expression, including their roles in both nuclear and cytoplasmic functions. However, the mechanisms regulating circRNA translation, elongation, and termination remain incompletely understood.

## 3 Functional Implications of Circrna Modulation by Natural Antioxidants

circRNA modulation by natural antioxidants involves structurally diverse compounds, including flavonoids, carotenoids, polyphenols, vitamins, terpenoids, and alkaloids, each with unique bioactive properties such as antioxidant, anti‐inflammatory, anticancer, and cardioprotective effects (Ramli, Cheriet, Posadino, et al. 2023, 2024; Mansour et al. 2024; Ramli, Cheriet, Thuan, et al. 2024; Posadino, Giordo, Pintus, et al. 2023; Posadino, Giordo, Ramli, et al. 2023; Shaito et al. 2023; Giordo et al. 2023; Hossain et al. 2022). These properties translate into circRNA regulation via mechanisms including miRNA sponging, RBP interaction, and modulation of transcriptional and post‐transcriptional processes (Giordo et al. 2022; Ramli, Cheriet, Posadino, et al. 2023; Ramli, Posadino, Giordo, et al. 2023).

This section categorizes antioxidants by chemical class and highlights representative compounds that modulate circRNA expression in specific disease contexts. This classification links the structural attributes of antioxidants to their modulatory mechanisms on circRNAs and their therapeutic potential.

## 4 Flavonoids

Flavonoids comprise a large group of polyphenolic compounds widely distributed in fruits, vegetables, and medicinal herbs. They include flavonols, flavones, flavanones, isoflavones, and catechins and are known for antioxidant, anti‐inflammatory, anti‐cancer, and cardioprotective properties (Anand David et al. 2016; Vollmannová et al. 2024). Among these, quercetin, a flavonol abundant in citrus fruits, onions, leafy greens, and berries—has garnered attention for its broad biological activities, including antioxidant (Song et al. 2020), anti‐inflammatory effects (Tian et al. 2021) anti‐diabetic activity (Ali et al. 2024), anti‐cancer and chemopreventive actions (Dogan 2022; Lotfi et al. 2023; Ward et al. 2018), neuroprotection (Sabogal‐Guáqueta et al. 2015), osteoprotection (Yuan et al. 2018), and cardioprotection (Albadrani et al. 2021). These diverse properties, along with emerging evidence of circRNA modulation, place quercetin at the center of interest in RNA‐based regulatory mechanisms.

### 4.1 Quercetin

*Quercetin* is a yellow flavonol that is abundant in green, leafy vegetables, citrus fruits, onions, and berries. It exhibits a broad spectrum of bioactivities, including antioxidant, anti‐inflammatory, anti‐diabetic, anti‐cancer, neuroprotective, osteoprotective, and cardioprotective effects (Albadrani et al. 2021; Ali et al. 2024; Anand David et al. 2016; Dogan 2022; Lotfi et al. 2023; Sabogal‐Guáqueta et al. 2015; Song et al. 2020; Tian et al. 2021; Vollmannová et al. 2024; Ward et al. 2018; Yuan et al. 2018).

*Quercetin* exerts osteoprotective effects by modulating the differentiation of mesenchymal stem cells (BMSCs). In ER‐α‐deficient BMSCs, it promotes osteogenesis by increasing ALP, COL1, and RUNX2 while suppressing adipogenesis via C/EBP‐α and PPAR‐γ regulation (Yuan et al. 2018; Li, Chen, et al. 2021). Bioinformatics analyses identified 136 circRNAs modulated by ER‐α, 120 by QUE, and 32 oppositely regulated by both (Li, Chen, et al. 2021). Several of these circRNAs participate in Wnt and Rap1 signaling, particularly targeting miR‐326‐5p, which upregulates osteogenic genes (Col1a1, Col5a1, Col18a1) and represses adipogenic ones (Hcav2, Zc3h12a, Sc125a35, Acad10), underscoring a circRNA‐mediated mechanism in lineage commitment (Li, Chen, et al. 2021).

In cancer, quercetin reverses drug resistance and suppresses progression via circRNA axes. In Palbociclib‐resistant breast cancer cells, quercetin induces circHIAT1, which sponges miR‐19a‐2p, restoring CADM2 and Palbociclib sensitivity. This axis was validated in xenograft models, where quercetin reduced tumor growth and EMT (Li et al. 2024). In prostate cancer, quercetin modulates radiotherapy resistance through ARv7‐mediated circRNA dysregulation. ARv7 upregulates circNHS, which acts as a sponge for miR‐512‐5p and enhances XRCC5 expression, thereby promoting DNA repair. Quercetin downregulates ARv7 and circNHS while increasing miR‐512‐5p, impairing repair and promoting apoptosis. The resulting suppression of tumor growth via the circNHS/miR‐512‐5p/XRCC5 pathway demonstrates quercetin's role in radiosensitization (Chen, Chou, et al. 2022; Senapati et al. 2023).

Quercetin also mitigates myocardial infarction (MI)‐induced remodeling by regulating circRNA. It improves cardiac function and reduces infarct size (~21%) in rats (Albadrani et al. 2021; Farazi et al. 2024). Mechanistically, quercetin downregulates circPAN3 and PTEN while upregulating miR‐221, which targets PTEN. Since circPAN3 binds miR‐221 and promotes fibrosis and apoptosis, quercetin's modulation of the circPAN3/miR‐221/PTEN axis contributes to cardioprotection (Li et al. 2020; Mahmoud et al. 2019; Zhou et al. 2019). Furthermore, quercetin enhances PI3K/AKT signaling and inhibits caspase‐3, fostering cardiomyocyte survival. These findings demonstrate how quercetin regulates cell fate and repair mechanisms through the interaction of circRNA, miRNA, and mRNA axes in bone, cancer, and cardiac contexts.

### 4.2 Kaempferol

Kaempferol, a dietary flavonol found in tea, broccoli, grapes, and *Ginkgo biloba* , exhibits a wide range of biological effects, including anti‐cancer, anti‐inflammatory, cardioprotective, and neuroprotective activities (Bian et al. 2022; Che et al. 2017; Choi et al. 2015; Da et al. 2019; Dang et al. 2015; Devi et al. 2015; Kadioglu et al. 2015; Kouhestani et al. 2018; Ren et al. 2019; Zhou et al. 2023). Its endothelial protective role in atherosclerosis has been linked to the circRNA–miRNA–mRNA regulatory axis. In HUVECs exposed to oxidized LDL (ox‐LDL), Kaempferol treatment enhanced cell viability and downregulated circNOL12 (Li, Hao, et al. 2021). Silencing circNOL12 decreased inflammation (TNF‐α, IL‐6, IL‐1β), intracellular ROS, and apoptosis markers (Bax, caspase‐3), while increasing the anti‐apoptotic Bcl‐2 (Li, Hao, et al. 2021).

Mechanistically, kaempferol upregulated miR‐6873‐3p, which targets FGF receptor substrate 2 (FRS2), a mediator of pro‐atherogenic fibroblast growth factor signaling (Goetz and Mohammadi 2013; Qi and Xin 2019). Overexpression of circNOL12 reversed this effect by sponging miR‐6873‐3p, restoring FRS2 levels. This circNOL12/miR‐6873‐3p/FRS2 axis highlights kaempferol's capacity to modulate oxidative and inflammatory responses through circRNA‐mediated signaling.

In colorectal cancer (CRC), kaempferol inhibits metastasis by disrupting the circRNA–miRNA–epigenetic interface. Specifically, it downregulates JMJD2C (KDM4C), a β‐catenin coactivator and EMT driver, by upregulating miR‐205‐5p, which directly targets JMJD2C (Li and Jiang 2017; Pu et al. 2024; Shang et al. 2017). CRC patient data reveal that circ_0000345, overexpressed in metastatic tumors, sponges miR‐205‐5p and facilitates JMJD2C/β‐catenin signaling (Pu et al. 2024). Kaempferol suppresses the expression of circ_0000345, potentially via RBPs involved in its biogenesis (e.g., HNRNPK/L), thereby inhibiting the migration and invasion of CRC cells.

In vivo, kaempferol significantly reduces metastatic tumor burden in CRC lung metastasis models, matching the efficacy of capecitabine (Pu et al. 2024). These findings establish a mechanistic framework in which kaempferol inhibits cancer progression through the circ_0000345/miR‐205‐5p/JMJD2C/β‐catenin pathway, highlighting the therapeutic potential of flavonols in regulating the crosstalk between circRNA and miRNA in tumor metastasis.

### 4.3 Baicalein

Baicalein, a flavone from *Scutellaria baicalensis*, exerts anti‐cancer, anti‐inflammatory, neuroprotective, and cardioprotective effects (Chai et al. 2017; Cheng et al. 2018; Cui et al. 2014; Guo et al. 2015; Liu, Li, et al. 2021; Mu et al. 2016; Sowndhararajan et al. 2018; Yu, Chen, et al. 2021; Yu et al. 2018; Zhao et al. 2016). In cervical cancer (CC) cells, baicalein induces G_0_/G_1_ arrest and apoptosis by regulating a circRNA–miRNA axis. It upregulates circHIAT1 while downregulating miR‐19a‐3p, an oncogenic miRNA (Hu et al. 2021). Disruption of this axis, by miR‐19a‐3p overexpression or circHIAT1 knockdown, abolishes baicalein's anti‐proliferative effects, confirming that tumor suppression occurs via circHIAT1‐mediated miR‐19a‐3p inhibition and downstream suppression of AKT/mTOR signaling (Glaviano et al. 2023; Hu et al. 2021). In xenograft models, baicalein reduced tumor growth, correlating with elevated circHIAT1 and depletion of miR‐19a‐3p (Hu et al. 2021).

Baicalein also downregulates circMYH9 in CRC, enhancing miR‐761 availability and suppressing HDGF, a pro‐oncogenic factor that drives proliferation and β‐catenin/Wnt signaling (Lian et al. 2015; Liu, Liu, et al. 2021; Zhang, Liu, et al. 2021). This circMYH9/miR‐761/HDGF axis mediates baicalein's inhibition of CRC cell migration, invasion, and tumorigenesis both in vitro and in vivo (Zhang, Liu, et al. 2021). These findings reinforce baicalein's multi‐targeted anticancer mechanisms, which involve reprogramming circRNA–miRNA networks to suppress oncogenic signaling cascades.

### 4.4 Luteolin

Luteolin, a flavone abundant in foods such as onions, carrots, and various medicinal herbs, exhibits neuroprotective, cardioprotective, anti‐inflammatory, and anticancer properties (Aziz et al. 2018; Imran et al. 2019; Luo et al. 2017; Nabavi et al. 2015; Ntalouka and Tsirivakou 2023). In non‐small cell lung cancer (NSCLC), luteolin suppresses tumor cell proliferation, migration, and invasion while inducing apoptosis through mitochondrial pathways, including Bax and caspase‐3 activation, as well as Bcl‐2 downregulation (Meng et al. 2016; Zheng et al. 2023).

Luteolin's anti‐NSCLC activity is mediated by the downregulation of circ_0000190 and the upregulation of miR‐130a‐3p. Normally, circ_0000190 sponges miR‐130a‐3p, facilitating Notch1 pathway activation (Notch1, Hes1, VEGF), which supports tumor progression. Luteolin treatment disrupts this axis by releasing miR‐130a‐3p, which suppresses Notch1 signaling and thereby inhibits tumor growth and angiogenesis. Overexpression of circ_0000190 reverses luteolin's effects, confirming the mechanistic importance of the circ_0000190/miR‐130a‐3p/Notch1 pathway (Zheng et al. 2023). Xenograft models further support these findings, linking luteolin administration to a reduced tumor burden and decreased expression of circ_0000190.

**FIGURE 2:**
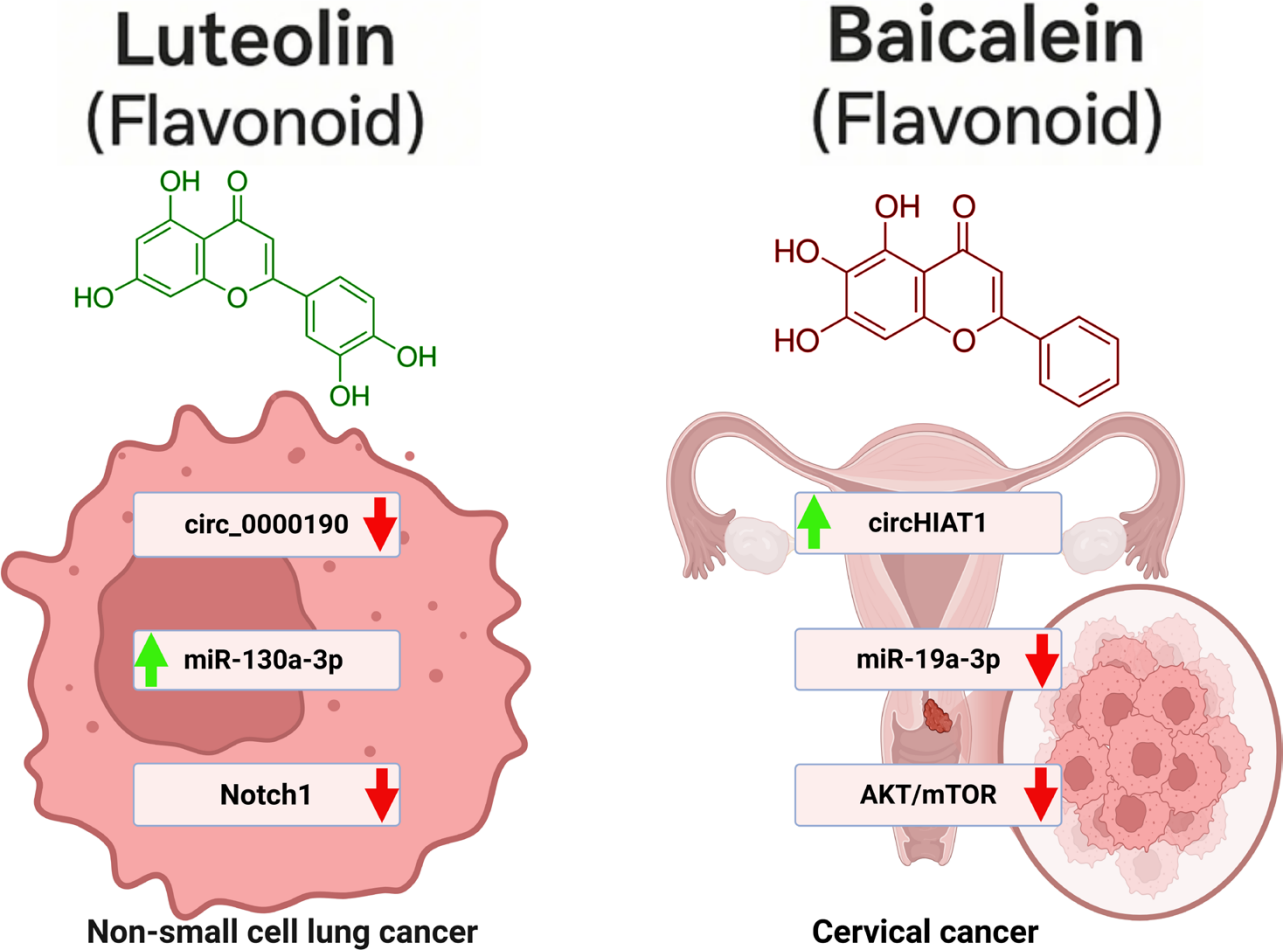
Schematic representation of the proposed molecular mechanisms by which Luteolin and Baicalein exert their anticancer effects. In non‐small cell lung cancer, Luteolin's activity is linked to the downregulation of circ_0000190, the concurrent upregulation of miR‐130a‐3p, and subsequent inhibition of Notch1. Conversely, in cervical cancer, Baicalein promotes the expression of circHIAT1, leading to the downregulation of miR‐19a‐3p and the suppression of the AKT/mTOR signaling pathway. These interactions highlight the therapeutic potential of flavonoids in targeting oncogenic signaling pathways regulated by circRNA.

Additionally, extracts from *Scutellaria barbata* and *Oldenlandia diffusa*, rich in luteolin and apigenin, inhibit the proliferation and migration of hepatocellular carcinoma (HCC) cells by inducing apoptosis and modulating the circRNA profiles (Yang et al. 2021; Yao et al. 2023). Gene ontology analyses revealed differential expression of hundreds of circRNAs involved in metabolic pathways, organelle function, and key signaling cascades, including mTOR, AMPK, and Wnt (Yang et al. 2021). These findings suggest that luteolin's anticancer effects are mediated by circRNA‐regulated processes, underscoring its relevance in circRNA‐targeted therapeutic strategies. Figure 2 illustrates the circRNA‐mediated molecular pathways through which luteolin and baicalein exert their anticancer effects in non‐small cell lung cancer and CC, respectively, highlighting the disruption of key oncogenic signaling cascades via modulation of specific circRNA–miRNA axes.

### 4.5 Diosmetin

Diosmetin, a flavone found in citrus fruits and medicinal herbs, displays antioxidant, anti‐inflammatory, and anticancer activities (Garg et al. 2022). In a dextran sulfate sodium (DSS)‐induced colitis mouse model, diosmetin ameliorates disease severity by reducing inflammation, oxidative stress, and epithelial barrier dysfunction (Liu, Fu, et al. 2023; Shalkami et al. 2018; Yu and Liu 2021). These effects correlate with increased expression of circSirt1 and its protein‐coding counterpart Sirt1, along with activation of the Nrf2/HO‐1 pathway and suppression of NF‐κB acetylation (Li et al. 2022).

CircSirt1 appears central to diosmetin's protective actions: its silencing negates the anti‐inflammatory and antioxidant effects, while diosmetin can rescue these impairments. Mechanistically, circSirt1 enhances Sirt1 expression, which promotes Nrf2 signaling—key to antioxidant defense—and represses pro‐inflammatory NF‐κB signaling. Diosmetin also modulates the gut microbiota, increasing beneficial bacterial populations that contribute to mucosal integrity (Li et al. 2022). Thus, diosmetin exerts multi‐layered protection in colitis through the circSirt1/Sirt1 axis, highlighting circRNA‐dependent modulation of oxidative and inflammatory networks.

### 4.6 Hesperetin

Hesperetin, a flavanone derived from hesperidin in citrus fruits, exhibits hepatoprotective and anti‐inflammatory properties. In a lipopolysaccharide/D‐GalN‐induced acute liver injury model, a hesperetin derivative (HD‐2a) provided protection through the regulation of circRNA (Sun et al. 2024). Injury was associated with the upregulation of circDcbld2 in hepatic tissue and macrophages. Silencing circDcbld2 mitigated liver damage, reduced the expression of inflammatory cytokines (IL‐1β, TNF‐α, MCP‐1), and improved tissue architecture (Sun et al. 2024).

HD‐2a treatment downregulated circDcbld2 both in vitro and in vivo, concurrently suppressing JAK2/STAT3 pathway activation, a major driver of inflammation. Restoration of circDcbld2 reversed these protective effects, indicating it functions upstream of JAK2/STAT3 signaling (Sun et al. 2024). Thus, hesperetin's hepatoprotective effects involve the inhibition of circDcbld2, positioning this circRNA as a key molecular node in the regulation of acute liver inflammation and oxidative stress by flavonoids.

### 4.7 Eriodictyol

Eriodictyol, a naturally occurring flavanone, exerts potent anticancer activity in breast cancer models without affecting normal epithelial cells (He et al. 2022). Eriodictyol reduces viability and induces apoptosis in MCF‐7 and MDA‐MB‐231 cells by increasing the cleavage of caspase‐3 and PARP. It also inhibits carcinogenesis in non‐tumorigenic MCF10A cells exposed to NNK or B[a]P by suppressing growth factor dependence and anchorage‐independent growth (He et al. 2022).

In vivo, eriodictyol reduces mammary tumor formation (~50%) in MNU‐treated rats without adverse effects. Mechanistically, eriodictyol inhibits the PI3K/AKT pathway by downregulating the phosphorylation of PI3K, AKT, and c‐Myc, and suppresses the expression of circ_0007503 (He et al. 2022). This circRNA acts as a sponge for tumor‐suppressive miRNAs; its overexpression reverses eriodictyol's effects by restoring PI3K/AKT activation and reducing apoptosis (He et al. 2022). Thus, eriodictyol's anti‐tumor function relies on the inhibition of circ_0007503 and subsequent suppression of the PI3K/AKT pathway, highlighting its value in circRNA‐targeted cancer therapy.

### 4.8 Naringin

Naringin, a flavanone glycoside abundant in grapefruit and other citrus fruits, has been investigated for its effects on ovarian hyperstimulation syndrome (OHSS), a condition characterized by an exaggerated ovarian response to hormonal stimulation. In OHSS rat models, high‐dose naringin improved fertility parameters and endometrial receptivity by modulating the expression of circRNA (Yuan et al. 2024). Specifically, it downregulated rno_circ_008140, which is abnormally elevated in OHSS.

Bioinformatics analysis identified circ_008140 as a sponge for 24 miRNAs, including miR‐323‐5p and miR‐99a‐5p, which target genes linked to endometrial receptivity (e.g., Abcc4, Rps6ka5) (Yuan et al. 2024). Naringin's suppression of circ_008140 may free these miRNAs to regulate gene networks, enhancing uterine receptivity and implantation success (Yuan et al. 2024). Although primarily reproductive, this model highlights the systemic reach of flavonoid–circRNA interactions across physiological contexts.

### 4.9 Catechins

Catechins, including epigallocatechin gallate (EGCG), are flavanols found in green tea with anti‐cancer and cardioprotective properties. In melanoma cells, a green tea polyphenol mixture (GTP) reduced proliferation, migration, and EMT while promoting apoptosis via the circ_MITF/miR‐30e‐3p/HDAC2 axis (Wu, Wei, et al. 2022). GTP suppressed circ_MITF, thereby freeing miR‐30e‐3p to inhibit HDAC2 and restore tumor‐suppressive signaling. This effect translated in vivo with reduced tumor burden and EMT markers.

In hepatic tissue, EGCG modulated 35 circRNAs, including circ_011775, which downregulates pro‐fibrotic and inflammatory genes (e.g., Col1a1, Il6), affecting AGE‐RAGE signaling in diabetic complications (Yoshitomi et al. 2024). In diabetic nephropathy models, catechin‐rich extracts altered renal circRNA profiles, with 30 differentially expressed circRNAs linked to miRNA targets enriched in insulin and AMPK pathways (Chen, Zhu, et al. 2022).

In RCC, circSPIRE1, which is downregulated in tumors, was shown to suppress metastasis through the ELAVL1‐mediated stabilization of tumor suppressors (GLANT3, QKI) and the reinforcement of endothelial barriers via exosomal transfer (Shu et al. 2023). Nanoparticle delivery of circSPIRE1 plasmids enhanced therapeutic efficacy, underscoring the translational value of catechin–circRNA strategies in metastatic cancer.

### 4.10 Genistein

Genistein, an isoflavone derived from soybeans, exhibits potent anticancer effects by targeting ncRNAs, including circRNAs. In NSCLC, genistein suppresses tumor growth by downregulating circ_0031250, which acts as a sponge for miR‐873‐5p and promotes the expression of FOXM1, a transcription factor that drives proliferation and survival (Yu, Xing, et al. 2021). Silencing circ_0031250 enhances the efficacy of genistein in reducing cell proliferation, invasion, and tumor growth in vitro and in xenografts.

In pancreatic cancer, genistein—alone or in combination with curcumin or quercetin—modulates circRNAs, such as circHIPK3 and circ_0000284, which are implicated in chemoresistance (Xie et al. 2022). It may sensitize tumor cells to chemotherapy by reprogramming resistance‐associated ncRNA networks.

Genistein also influences Hedgehog and Hippo signaling pathways, which are regulated by ncRNAs, including circRNAs, that affect GLI1 and YAP/TEAD activity (Sharma et al. 2022). These interactions support its multitarget potential in disrupting transcriptional and post‐transcriptional oncogenic circuits. As part of the dietary polyphenol class, genistein modulates ROS‐scavenger‐triggered anticancer pathways (RSTAPs) by regulating ncRNAs, suggesting a dual action through redox control and circRNA‐mediated gene expression (Hayakawa et al. 2022).

### 4.11 Anthocyanins

Anthocyanins are flavonoids responsible for red to blue pigmentation in many fruits and vegetables, with recognized antioxidant, anti‐inflammatory, and anticancer activities (Posadino, Giordo, Ramli, et al. 2023). Cyanidin‐3‐glucoside (C3G), found in berries and *Morus alba* L., exhibits circRNA‐mediated hepatoprotective effects in HCC.

In a rat model of DEN/2‐AAF–induced liver cancer, C3G preserved liver architecture and function while modulating the circ_0001345/miR‐106b/ATG16L1 axis (Zabady et al. 2022). Circ_0001345 sponges miR‐106b, a negative regulator of ATG16L1, a gene essential for autophagy. C3G downregulates circ_0001345, allowing miR‐106b to suppress ATG16L1 and restore autophagic balance (Zabady et al. 2022). This highlights a novel tumor‐suppressive mechanism in which dietary anthocyanins regulate cancer‐relevant processes via circRNA–miRNA interactions.

## 5 Polyphenolic Antioxidants

### 5.1 Phenolic Acids

Phenolic acids, classified into hydroxybenzoic and hydroxycinnamic acids, are abundant in plant‐based foods and possess strong antioxidant and anti‐inflammatory properties. Recent studies have highlighted their capacity to regulate gene expression through ncRNAs, including circRNAs, which are involved in redox responses, apoptosis, and autophagy.

Gallic acid, a hydroxybenzoic acid, modulates complex regulatory networks involving miRNAs, lncRNAs, and circRNAs. In CC cells, gallic acid reversed oncogenic gene signatures (e.g., CDC20, DLGAP5, KIF20A) and altered the expression of 42 circRNAs implicated in an lncRNA/circRNA–miRNA–mRNA axis, highlighting its potential to reprogram circRNA‐dependent transcriptional networks (You et al. 2025).

In a rat model of premature ovarian insufficiency (POI), BSNXD—a traditional formula enriched in gallic acid restored—ovarian structure and redox balance by modulating circRNA and mRNA expression. A key circuit involved rno_circRNA_012284, which sponges miR‐760‐3p to enhance HBEGF expression and reduce ROS in granulosa cells. Silencing rno_circRNA_012284 abolished these effects, confirming its mechanistic role (Xu et al. 2024).

Within hydroxycinnamic acids, caffeic acid phenethyl ester, a propolis‐derived compound, protects hepatocytes from cadmium toxicity by inhibiting autophagy‐dependent circRNA. Caffeic acid phenethyl ester downregulated hsa_circ_0040768, a positive regulator of MAP1LC3B, and its overexpression reversed caffeic acid phenethyl ester's cytoprotective effect (Hao et al. 2020). These findings position phenolic acids as modulators of circRNA‐guided redox and cell stress pathways.

## 6 Non‐Flavonoid Polyphenols

Beyond the flavonoids, many other polyphenolic compounds from dietary sources exhibit regulatory effects on the expression and function of circRNA. Polyphenols, as a class (including phenolic acids, stilbenes, and lignans), are well known for their antioxidant and anti‐inflammatory activities, which contribute to the prevention of cardiovascular and liver diseases, among others. Two of the most prominent polyphenols studied in this context are curcumin and resveratrol.

### 6.1 Curcumin

Curcumin, a turmeric‐derived polyphenol, exerts antioxidant, anti‐inflammatory, and anticancer effects via ncRNA modulation (Hu et al. 2024; Joshi et al. 2021). In renal fibrosis, curcumin suppresses apoptosis and fibrosis through the circ_0008925/miR‐204‐5p/IL6ST axis. Silencing circ_0008925 or restoring miR‐204‐5p abolished its anti‐fibrotic actions (An et al. 2025).

In fertility and neuroprotection models, curcumin mitigates nanoparticle toxicity and Parkinson's‐related apoptosis by regulating miR‐21, miR‐211, and circRNA0001518 expression, modulating Bax/Bcl‐2 balance (Khosravi et al. 2023; Paskeh et al. 2024). In CRC, curcumin inhibits EMT and tumor growth via the circHN1/miR‐302a‐3p/PIK3R3 axis (Wu, Zhao, et al. 2022).

In NSCLC, curcumin suppresses ferroptosis resistance by downregulating circFOXP1, which acts as a sponge for miR‐520a‐5p and upregulates SLC7A11 (Wang, Xie, and Malhotra 2021). In ovarian and liver cancer, it modulates circ‐PLEKHM3/miR‐320a/SMG1 and circ_0078710/miR‐378b/PRIM2 axes, respectively, enhancing apoptosis and impairing tumor progression (Chen, Guo, et al. 2022; Sun and Fang 2021).

Additional mechanisms include the circ‐FNDC3B/miR‐138‐5p/IGF2 axis in renal carcinoma and the circ‐PRKCA/miR‐384/ITGB1 network in NSCLC (Xu et al. 2021). In nasopharyngeal carcinoma, curcumin enhances radiosensitivity via the circRNA_102115/miR‐335‐3p/MAPK1 circuit (Zhu et al. 2020). These findings illustrate curcumin's multi‐targeted modulation of circRNA–miRNA–mRNA networks in diverse disease contexts.

### 6.2 Resveratrol

Resveratrol, a non‐flavonoid polyphenol, modulates diverse classes of ncRNAs, including circRNAs, implicated in oxidative stress, apoptosis, proliferation, and neuroprotection (Giordo et al. 2022; Ramli, Cheriet, Posadino, et al. 2023; Ramli, Posadino, Giordo, et al. 2023).

In porcine ovarian granulosa cells, resveratrol significantly altered the expression of over 10,000 circRNAs, with enrichment in redox‐ and apoptosis‐related pathways, such as the mTOR and AMPK pathways. Novel circRNAs (e.g., circ_0012954, circ_0004762) displayed strong connectivity in miRNA networks, suggesting functional roles as sponges in reproductive regulation (Zhang, Ye, et al. 2024).

In ovarian cancer, resveratrol inhibited proliferation and promoted apoptosis, which is linked to the modulation of the circRNA–miRNA–mRNA network. RNA‐seq revealed hundreds of differentially expressed circRNAs and miRNAs influencing key oncogenic pathways (Zhu et al. 2024). Similarly, resveratrol exhibited neuroprotective effects in Alzheimer's models by downregulating circ_0050263, which normally sponges miR‐361‐3p, thereby promoting PDE4A expression. This axis was crucial in mitigating neurotoxicity, apoptosis, and endoplasmic reticulum (ER) stress (Zhang, Chen, et al. 2023).

Across systems, resveratrol and other polyphenols, such as curcumin, genistein, and quercetin, exert ROS‐scavenger‐triggered anticancer effects (RSTAPs) by regulating tumor‐suppressive and oncogenic ncRNAs. Their redox behavior depends on concentration and context, highlighting the complex interplay between ROS buffering and modulation of the circRNA network (Hayakawa et al. 2022). Resveratrol thus exemplifies the therapeutic potential of polyphenols in orchestrating ncRNA‐directed gene regulation in oxidative stress‐related pathologies.

## 7 Carotenoids

Carotenoids are C_40_ tetraterpenes responsible for the pigmentation of fruits and vegetables such as carrots and tomatoes. Their antioxidant capacity stems from their conjugated double bonds, enabling them to quench singlet oxygen and peroxyl radicals, thereby protecting lipids, proteins, and DNA from oxidative damage (Flieger et al. 2024; Liao et al. 2022; Michalak 2022). Beyond radical scavenging, carotenoids like β‐carotene and lycopene modulate redox‐sensitive signaling pathways involved in cellular stress responses.

Astaxanthin, a xanthophyll carotenoid found in microalgae and seafood, exhibits antioxidant, anti‐inflammatory, and cardio‐ and neuroprotective activities (Ambati et al. 2014; Baralic et al. 2015; Chintong et al. 2019; Fakhri et al. 2018; Farruggia et al. 2018; Sun et al. 2020; Wang et al. 2019; Zaafan and Abdelhamid 2021; Zuluaga et al. 2018). Astaxanthin exhibits various beneficial biological activities, including antioxidant, anti‐atherosclerotic, anti‐inflammatory, anti‐tumor, anti‐obesity, and cardioprotective properties.

Astaxanthin modulates the expression of circRNA in multiple disease models. In atherosclerosis, it enhances cholesterol efflux via the circTTP2/miR‐3073b‐5p/ABCA1 axis. Astaxanthin also upregulates circUGGT2, circPCMTD1, and circBRWD1 in ox‐LDL‐treated macrophages, sponging miRNAs (miR‐30a‐3p, miR‐139‐5p, miR‐3918) and promoting ABCA1/ABCG1/SR‐B1 expression (Liang et al. 2024; Liu, Wei, et al. 2021; Zhang, Zhuo, et al. 2024; Zhang, Qiu, et al. 2023).

In cardiac hypertrophy, astaxanthin reduces the expression of hypertrophic markers (ANP, BNP, β‐MHC) via the circ_0078450/miR‐338‐3p/GATA4 axis. Astaxanthin also downregulates METTL‐2, limiting m6A methylation of circ_0078450 and GATA4 expression, suggesting epigenetic control of circRNA biogenesis (Zhang, Zhuo, et al. 2024).

Reduced GATA4 expression was regulated by inhibiting the expression of circ_0078450 and protecting miR‐139‐5p in osteoarthritis. Astaxanthin restores chondrocyte redox balance by modulating the circHP1BP3/miR‐139‐5p/SOD1 signaling pathway. This axis reduces inflammatory cytokines (IL‐6, TNF‐α), ROS, and apoptotic markers, while promoting the expression of collagen II and Bcl‐2 (Liang et al. 2024).

**FIGURE 3:**
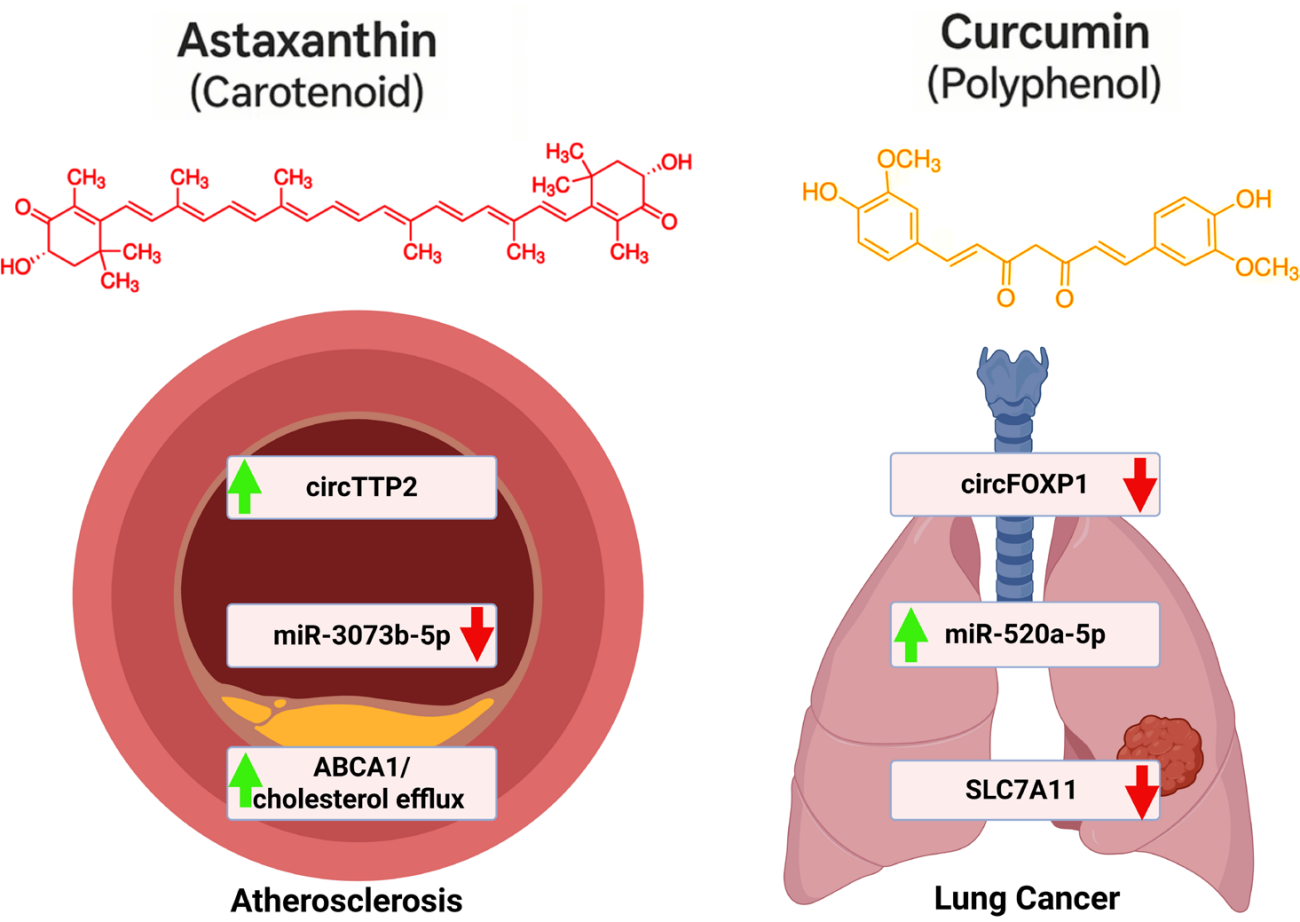
Illustrative representation of the molecular mechanisms underlying the therapeutic effects of Astaxanthin and Curcumin. In atherosclerosis, Astaxanthin enhances cholesterol efflux by upregulating circTTP2 and subsequently suppressing miR‐3073b‐5p, leading to increased ABCA1 expression. In lung cancer, Curcumin inhibits tumor progression by downregulating circFOXP1, which results in the upregulation of miR‐520a‐5p and a decrease in SLC7A11 expression. These interactions exemplify redox‐active nutraceuticals targeting circRNA‐mediated pathways.

Although less explored, other carotenoids may affect circRNA regulation in metabolic tissues. In obese mice, carotenoid‐rich algal extracts reduced oxidative stress and inflammation, and activated AMPK, a pathway that plausibly intersects with circRNA‐regulated metabolic signaling (Kurniawan et al. 2023). These findings suggest that carotenoids exert their biological effects, in part, by modulating circRNA‐guided redox and metabolic networks. Figure 3 summarizes the circRNA‐dependent molecular pathways through which astaxanthin and curcumin exert their protective effects in atherosclerosis and cancer, respectively, highlighting the modulation of key miRNA targets and downstream signaling cascades involved in cholesterol metabolism and tumor progression.

## 8 Vitamins A, C, and E

Vitamins A, C, and E are essential antioxidants with distinct roles in redox regulation. Vitamin A (retinol/ATRA) influences cell differentiation and immune function; vitamin C scavenges ROS and regenerates glutathione; vitamin E protects membranes from lipid peroxidation (Carr and Maggini 2017; Chen and Khillan 2010; Kaźmierczak‐Barańska et al. 2020; Khillan 2014; Michalak 2022; Padayatty and Levine 2016; Raverdeau and Mills 2014; Tan et al. 2018). Recent studies show their ability to modulate circRNA networks involved in oxidative stress‐related pathologies.

In a high‐fat diet mouse model, supplementation with vitamins C, E, B12, selenium, and glutathione restored sperm motility and reduced 4‐HNE levels. These effects were associated with the re‐establishment of FUS‐dependent circRNA networks (ceRNETs), indicating their involvement in redox and fertility regulation (Mele et al. 2023). In testicular hyperthermia models, curcumin with vitamins D and E, plus Fe_2_O_3_ and MnO_2_ nanoparticles, reduced apoptosis and modulated the expression of Bcl‐2, miR‐21, and circRNA0001518 (Paskeh et al. 2024). Similar protective effects were observed in a Parkinson's disease model treated with vitamin E and TiO_2_ nanoparticles, which improved neuronal viability and circRNA0001518 expression (Jamali et al. 2022).

Vitamin A deficiency (VAD) disrupted the expression of 70 circRNAs in embryonic rat tissues, altering Wnt, PI3K‐AKT, FoxO, and mTOR signaling pathways—pathways critical for somite development. Affected circRNA–miRNA–mRNA networks highlight the role of circRNAs in embryonic patterning and congenital scoliosis (Chen et al. 2018). In acute promyelocytic leukemia, ATRA induced differentiation and altered 508 circRNAs, including circ‐HIPK2, which sponges miR‐124‐3p to regulate MAPK1. Circ‐HIPK2 was downregulated in APL patients, suggesting its potential as a diagnostic marker (Li, Ma, et al. 2018).

Finally, vitamin E prevents ferroptosis—a lipid peroxidation–driven form of cell death—by modulating redox balance and potentially circRNA expression. circRNAs have emerged as candidate regulators of ferroptosis‐relevant genes, implicating vitamins in circRNA‐mediated cytoprotection across diverse disease states (Ensoy et al. 2023). Collectively, these studies highlight the therapeutic potential of antioxidant supplementation, particularly vitamins C and E, in regulating circRNAs and enhancing cellular resilience against oxidative stress‐induced damage across various models.

## 9 Terpenoids

Terpenoids (isoprenoids) constitute a vast class of bioactive plant compounds, including monoterpenes, sesquiterpenes, and triterpenes, many of which display antioxidant and anti‐inflammatory properties. Among them, ginsenosides—triterpenoid saponins from *Panax ginseng* —exert cardioprotective effects. Ginsenoside Rb1, for example, reduces infarct size and improves cardiac function by limiting oxidative stress and inflammation in myocardial infarction models (Padmavathi and Ramkumar 2021).

Although circRNAs were not directly measured in these studies, the redox‐stabilizing effects of Rb1 and similar terpenoids likely help maintain physiological circRNA profiles in damaged tissues. Other terpenoids, such as ursolic acid, oleanolic acid, and glycyrrhizin, also modulate antioxidant enzymes and inflammatory mediators, potentially impacting circRNA biogenesis indirectly through redox‐sensitive transcription factors (e.g., Nrf2, NF‐κB) (Feng et al. 2018; Yaribeygi et al. 2019). While direct circRNA targets remain largely unexplored, the known effects of terpenoids on upstream regulators suggest a modulatory role in circRNA‐regulated stress response pathways.

## 10 Alkaloids

Alkaloids are nitrogenous plant‐derived compounds with diverse pharmacological activities, including antioxidant, anti‐inflammatory, and metabolic effects. Berberine, an isoquinoline alkaloid found in *Berberis* and *Coptis* species, exerts anti‐diabetic, anti‐cancer, and cardioprotective effects by activating AMPK and modulating JNK signaling—key upstream regulators of circRNA networks (Chang 2017).

In insulin‐sensitive tissues, berberine influences the expression of miRNA and lncRNA, which in turn can stabilize or destabilize circRNAs (Luo et al. 2021; Zhang, Yang, et al. 2021). Moreover, by lowering oxidative stress and hyperglycemia, berberine helps preserve circRNA homeostasis, potentially through enhanced RBP activity that stabilizes beneficial circRNAs (Cicero and Tartagni 2012; Wang, Sun, et al. 2021). For instance, in endothelial cells under oxidative stress, AMPK activation may enhance the expression of protective circRNA, thereby promoting vascular integrity.

Other alkaloids, such as diterpenoid alkaloids (e.g., those found in Aconitum), exert analgesic effects by modulating ion channels, which may indirectly influence gene expression and circRNA production in excitable tissues (Koch et al. 2025). Caffeine, a methylxanthine, has been shown to have antioxidant effects via Nrf2 pathway activation, suggesting that it may influence circRNAs linked to redox‐sensitive targets (Ikram et al. 2020; Zhang, Chen, et al. 2022). Similarly, capsaicin induces low‐level oxidative stress, which triggers adaptive antioxidant responses—a process that likely intersects with circRNA‐regulated mechanisms (Abdulaal et al. 2024; Hacioglu 2022; Zhang, Chen, et al. 2022).

**FIGURE 4:**
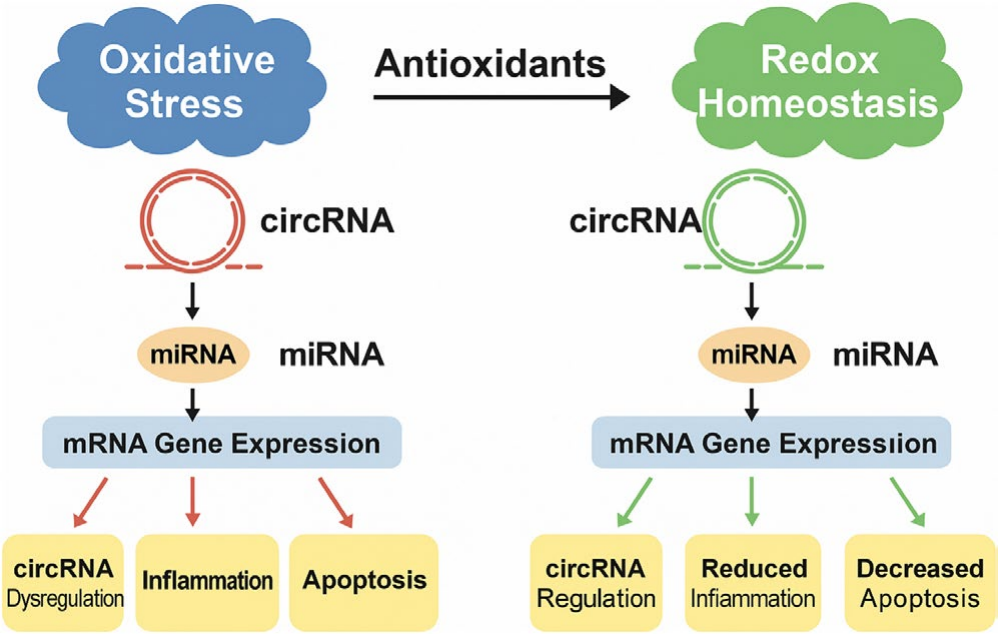
Schematic representation of the interplay between oxidative stress, antioxidants, and the circRNA‐miRNA‐mRNA regulatory axis in cellular homeostasis. The left panel illustrates how oxidative stress can disrupt the normal functioning of circRNAs, leading to the dysregulation of miRNA and mRNA expression, ultimately contributing to cellular inflammation and apoptosis. The right panel demonstrates how antioxidants counteract these effects, promoting redox homeostasis by re‐establishing healthy circRNA levels and functions, thereby leading to circRNA regulation, reduced inflammation, and decreased apoptosis through their influence on the circRNA‐miRNA‐mRNA pathway.

Although direct circRNA targets remain poorly defined, accumulating evidence—particularly for berberine—supports the hypothesis that alkaloids modulate circRNA function through upstream control of stress‐responsive transcription and RNA processing pathways. Figure 4 illustrates the central role of oxidative stress and antioxidant compounds in modulating circRNA‐miRNA‐mRNA networks. The key circRNA‐mediated molecular interactions described above are summarized in Table 2, which provides an overview of the specific antioxidant compounds, their circRNA targets, associated miRNA interactions, downstream pathways, and relevant disease models.

**TABLE 2:** Molecular mechanisms of antioxidant–circRNA interactions.

Compound (class)	circRNA modulated	Key miRNA involved	Affected pathway/target	Disease model/context
Baicalein (Flavonoid)	↑ circHIAT1	↓ miR‐19a‐3p	Upregulates PTEN, inhibits AKT/mTOR signaling	Cervical cancer cells and xenografts (tumor suppression) (Hu et al. 2021).
Luteolin (Flavonoid)	↓ circ_0000190	↑ miR‐130a‐3p	Downregulates Notch1 pathway (Hes1, VEGF)	Non‐small‐cell lung cancer (A549/H460 cells, xenograft) (Zheng et al. 2023).
Genistein (Isoflavone)	↓ circ_0031250	↑ miR‐873‐5p	Reduces FOXM1 expression (cell cycle/apoptosis genes)	Non‐small‐cell lung cancer (in vitro, mouse xenograft) (Yu, Xing, et al. 2021).
Curcumin (Polyphenol)	↓ circFOXP1	↑ miR‐520a‐5p	Inhibits SLC7A11, inducing ferroptotic cell death	Lung cancer (ferroptosis model in NSCLC cells) (Wang, Xie, and Malhotra 2021)
Resveratrol (Stilbene)	↓ circ_0050263	↑ miR‐361‐3p	De‐represses PDE4A, reducing neurotoxic stress	Alzheimer's disease model (Aβ‐treated neuronal cells) (Zhang, Chen, et al. 2023)
Cyanidin‐3‐O‐glucoside (Anthocyanin)	↑ circ_0001345	↓ miR‐106b‐5p	Upregulates ATG16L1, enhancing autophagy	Hepatocellular carcinoma (DEN/2‐AAF induced rat model) (Zabady et al. 2022).
Astaxanthin (Carotenoid)	↑ circTTP2	↓ miR‐3073b‐5p	Elevates ABCA1/G1, promoting cholesterol efflux	Atherosclerosis (ox‐LDL treated macrophages/foam cells) (Zhang, Qiu, et al. 2023)
All‐trans Retinoic Acid (Vitamin A)	↑ circ‐HIPK2	↓ miR‐124‐3p	Increases MAPK1 activity (promotes differentiation)	Acute promyelocytic leukemia (NB4 cells induced to differentiate) (Li, Ma, et al. 2018)

*Note:* Detailed examples of specific natural antioxidant compounds and their mechanistic circRNA interactions. Each entry lists the compound, the affected circRNA and its associated miRNA “sponge” interaction, the downstream pathway or gene influenced, and the disease or model in which this was demonstrated.

## 11 Therapeutic Integration and Personalized Medicine

### 11.1 Combining Natural Antioxidants With Other Therapeutic Modalities

Integrating natural antioxidants with conventional therapies enhances efficacy and reduces side effects (Aung et al. 2017). Compounds such as sulforaphane and resveratrol sensitize cancer cells to chemotherapy and protect healthy tissues from oxidative damage (M. Kaminski et al. 2012). This synergy may also apply to circRNA‐targeted strategies, improving RNA stability and function (Mondal et al. 2022).

**FIGURE 5:**
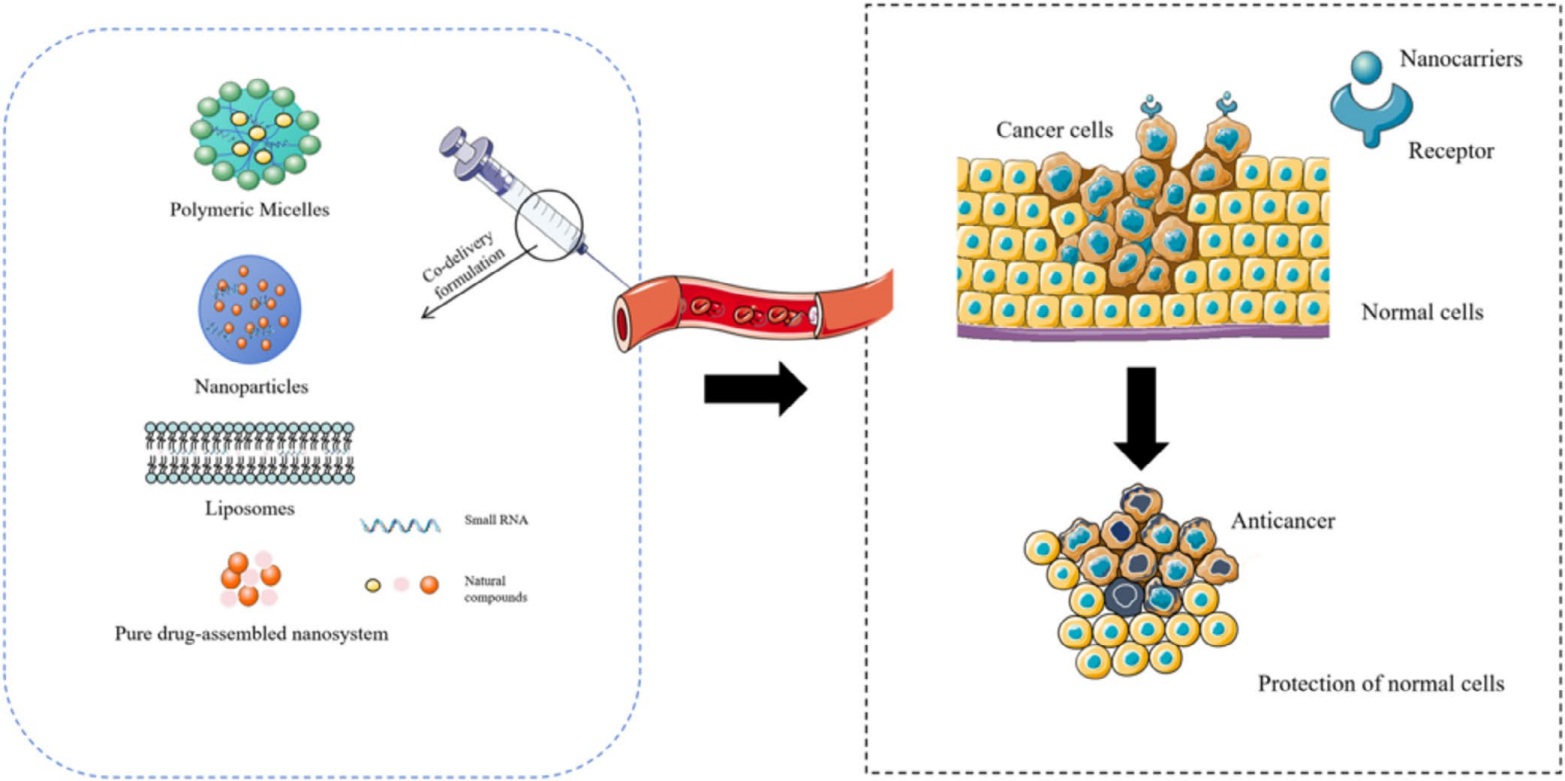
Schematic representation of advanced co‐delivery systems integrating natural antioxidants and circRNA‐targeted therapeutics. Conceptual illustration of a therapeutic co‐delivery strategy combining a natural antioxidant with a circRNA‐targeting intervention. Nanoparticle‐based carriers (liposomes, polymeric micelles, etc.) can be engineered to simultaneously encapsulate a bioactive antioxidant (e.g., curcumin, resveratrol) and a circRNA‐modulating agent (such as siRNA, antisense oligonucleotide, or shRNA expression vector targeting a disease‐related circRNA). After intravenous administration, these co‐delivery systems preferentially accumulate in diseased tissue (e.g., tumor, inflamed tissue) via enhanced permeability and retention effects. The antioxidant component reduces oxidative stress and inflammation locally, whereas the circRNA‐targeting component modulates pathological circRNA activity in cells. The combined action protects normal cells and yields enhanced therapeutic efficacy (e.g., inducing cancer cell death while sparing healthy cells). This multi‐modal approach leverages the synergism between antioxidants and gene‐silencing therapies, offering improved treatment outcomes in complex diseases.

Advanced drug delivery systems, including nanoparticles, liposomes, and hydrogels, enhance antioxidant bioavailability and facilitate targeted delivery to circRNA‐regulated pathways (Venkatas et al. 2022; Yang et al. 2020). Co‐delivery systems combining antioxidants with circRNA modulators (e.g., siRNAs, ASOs, expression vectors) enable simultaneous redox modulation and transcriptome reprogramming (Figure 5) (Chen et al. 2023). Furthermore, coupling antioxidants with Clustered Regularly Interspaced Short Palindromic Repeats—CRISPR‐associated proteins (CRISPR‐Cas) or RNAi technologies may enable the precise silencing or correction of pathogenic circRNAs (Huang et al. 2024; Rudenko et al. 2023), offering a multi‐pronged therapeutic strategy for complex diseases such as cancer and neurodegeneration.

## 12 Personalized Medicine Approaches

### 12.1 Genetic and Epigenetic Considerations

Interindividual variability in circRNA expression and antioxidant response supports a shift toward personalized therapies (Wu et al. 2023). Genetic variants can alter circRNA biogenesis, as shown in tissue‐specific profiling studies (Xu et al. 2017; Zeng et al. 2022). Epigenetic modifications also influence circRNA levels, as observed in oral squamous cell carcinoma (Li, Gong, et al. 2021; Mangiavacchi et al. 2023). These insights underscore the importance of considering both genomic and epigenomic landscapes when designing interventions based on antioxidant‐circRNA.

Additionally, polymorphisms in antioxidant enzymes modulate treatment outcomes. For instance, genetic variation affecting antioxidant metabolism may alter circRNA responses and therapeutic efficacy (Wu et al. 2024), highlighting the importance of integrating redox genetics in personalized RNA‐based strategies.

### 12.2 Tailoring Treatments Based on Individual circRNA Profiles

circRNA signatures have diagnostic and therapeutic relevance. Liquid biopsy studies demonstrate their utility in distinguishing disease states (Drula et al. 2024; Lee et al. 2023). circRNA–miRNA–mRNA networks also help identify actionable molecular targets, as shown in early‐onset schizophrenia (Li, Gong, et al. 2021). Though data on dose personalization are limited, disease‐specific circRNA expression patterns, as in vitiligo, support the feasibility of tailored circRNA‐guided therapy (Zhang, Hou, et al. 2022).

Incorporating multi‐omics data and circRNA profiling into clinical decision‐making could transform RNA therapeutics by aligning interventions with patient‐specific molecular landscapes (Goina et al. 2024; Vea et al. 2018).

## 13 Challenges and Limitations

Despite the mounting evidence supporting the regulatory role of natural antioxidants on circRNA networks, several challenges persist in both conceptual and technical domains:

- *Mechanistic ambiguity*: The precise mechanisms through which antioxidants modulate circRNA biogenesis remain unclear—whether via redox‐sensitive splicing, epigenetic remodeling, or upstream signaling (Nielsen et al. 2022; Szabo and Salzman 2016; Zhang et al. 2020).
- *Context dependence*: Antioxidant effects on circRNA pathways vary by cell type and disease state. A single compound may yield opposing circRNA–miRNA–mRNA responses in cancer vs. neurodegeneration; underscoring the need for system‐specific profiling.
- *Technical limitations*: circRNA detection is hampered by its circular topology. Standard RNA‐seq may misclassify or miss back‐splice junctions; necessitating the development of improved algorithms and long‐read sequencing tools (Digby et al. 2024; Dong et al. 2025).
- *In vivo validation gap*: Most findings are from in vitro studies. Functional circRNA manipulation via antisense oligos, CRISPR‐Cas9, or overexpression systems remains limited, restricting mechanistic and therapeutic insight (Wang, He, et al. 2021).
- *Bioavailability issues*: Many antioxidants show poor solubility and fast metabolism. Without optimized delivery (e.g., nanoparticles, liposomes) in vivo, the efficacy of circRNA targeting remains limited.
- *Precision medicine integration*: circRNA and antioxidant responses are influenced by individual genetics, epigenetics, and disease context. Personalization is crucial for therapeutic success.
- *Safety and regulatory concerns*: At high doses, some antioxidants act as pro‐oxidants, potentially disrupting the homeostasis of circRNA (Giordo et al. 2013; Pasciu et al. 2010; Posadino et al. 2013, 2017, 2019). Off‐target circRNA modulation may have adverse effects; necessitating rigorous toxicological profiling.

Overcoming these challenges is essential not only to unravel the full therapeutic potential of antioxidant–circRNA interactions, but also to pave the way for precision redox medicine in the era of RNA‐targeted interventions.

## 14 Future Research Directions

The convergence of natural antioxidant research with circRNA biology is yielding exciting insights with direct therapeutic implications. To translate these findings into effective clinical strategies, the following research directions are critical:

- *Mechanistic elucidation*: Clarify how antioxidants affect circRNA biogenesis and stability—via splicing factors, epigenetic pathways, or redox‐sensitive signaling.
- *Context‐specific profiling*: Map circRNA–antioxidant interactions across tissues, diseases, and dose ranges to define condition‐specific signatures.
- *Tool development*: Enhance circRNA annotation with long‐read sequencing and new algorithms (Digby et al. 2024; Dong et al. 2025)
- *High‐throughput screening*: Identify antioxidant compounds that modulate functional circRNAs at scale (Wang, He, et al. 2021).
- *In vivo validation*: Use transgenic models and genome‐editing platforms to verify therapeutic circRNA targets.
- *Theranostics*: Develop nanocarriers co‐delivering antioxidants and circRNA modulators to maximize specificity and efficacy.
- *Personalized strategies*: Integrate circRNA expression profiles and antioxidant responses into tailored nutritional or therapeutic interventions.
- *Translational vision*: Natural antioxidants should be viewed as programmable redox‐responsive regulators of circRNA networks, capable of restoring homeostatic gene expression with minimal toxicity. Indeed, the ultimate goal is to develop novel therapies that utilize natural antioxidants—or their synthetic analogs—to precisely reprogram circRNA networks, thereby restoring physiological gene regulation with minimal off‐target effects.

**FIGURE 6:**
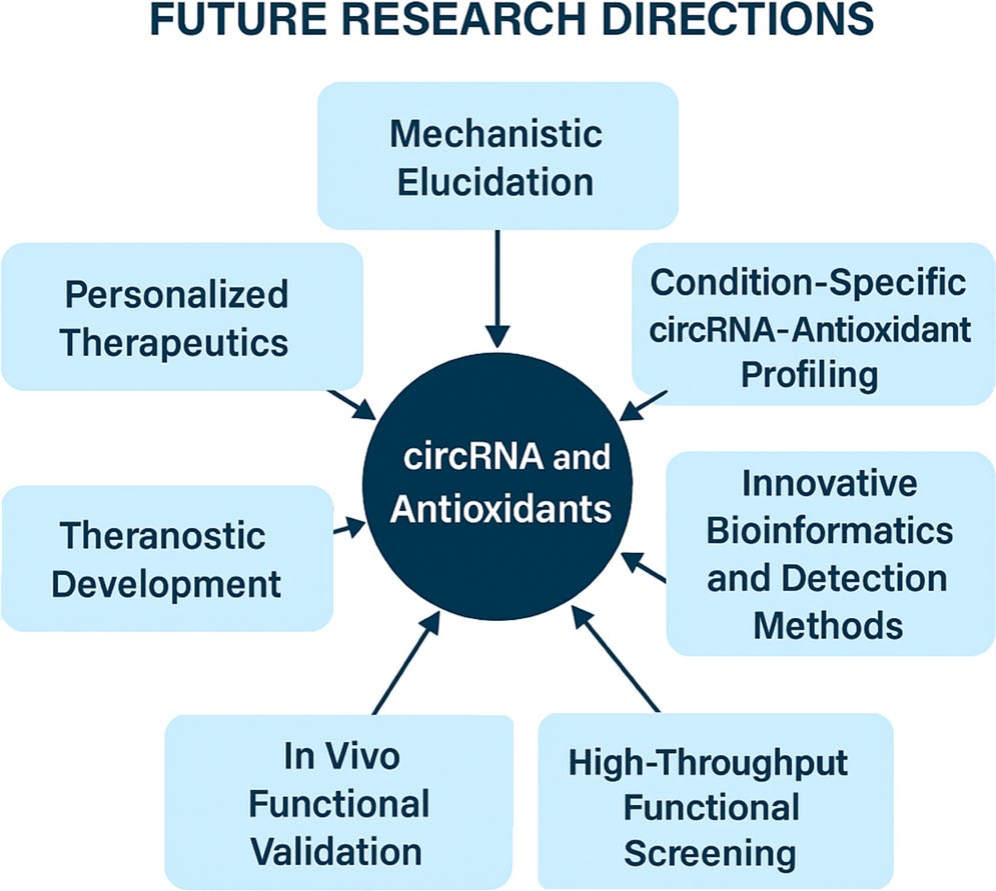
Key future research directions for harnessing the therapeutic potential of circRNAs in conjunction with antioxidants. This involves a comprehensive mechanistic understanding, the generation of condition‐specific circRNA‐antioxidant profiles to identify disease‐specific signatures, and the innovation of advanced bioinformatics and detection tools. Critical next steps also include high‐throughput functional screening and rigorous in vivo validation; ultimately aiming to translate these insights into personalized therapeutic strategies and integrated theranostic approaches.

As highlighted throughout this review, these compounds are more than passive ROS scavengers: they are dynamic epigenetic regulators with the potential to reshape cellular responses through circRNA‐mediated pathways. With continued interdisciplinary efforts bridging molecular biology, bioengineering, and clinical science, antioxidant–circRNA therapeutics may become a cornerstone of future disease‐modifying strategies. These strategic directions are summarized in Figure 6.

## 15 Conclusions

The mounting evidence reviewed herein underscores the pivotal role of natural antioxidants in modulating circRNA expression and function, revealing a novel axis of redox‐regulated gene regulation with significant therapeutic implications. Through diverse mechanisms—including miRNA sponging, RBP interaction, and modulation of epigenetic or transcriptional pathways—natural antioxidants can fine‐tune circRNA networks involved in oxidative stress, inflammation, apoptosis, and cellular differentiation across a broad spectrum of diseases.

**FIGURE 7:**
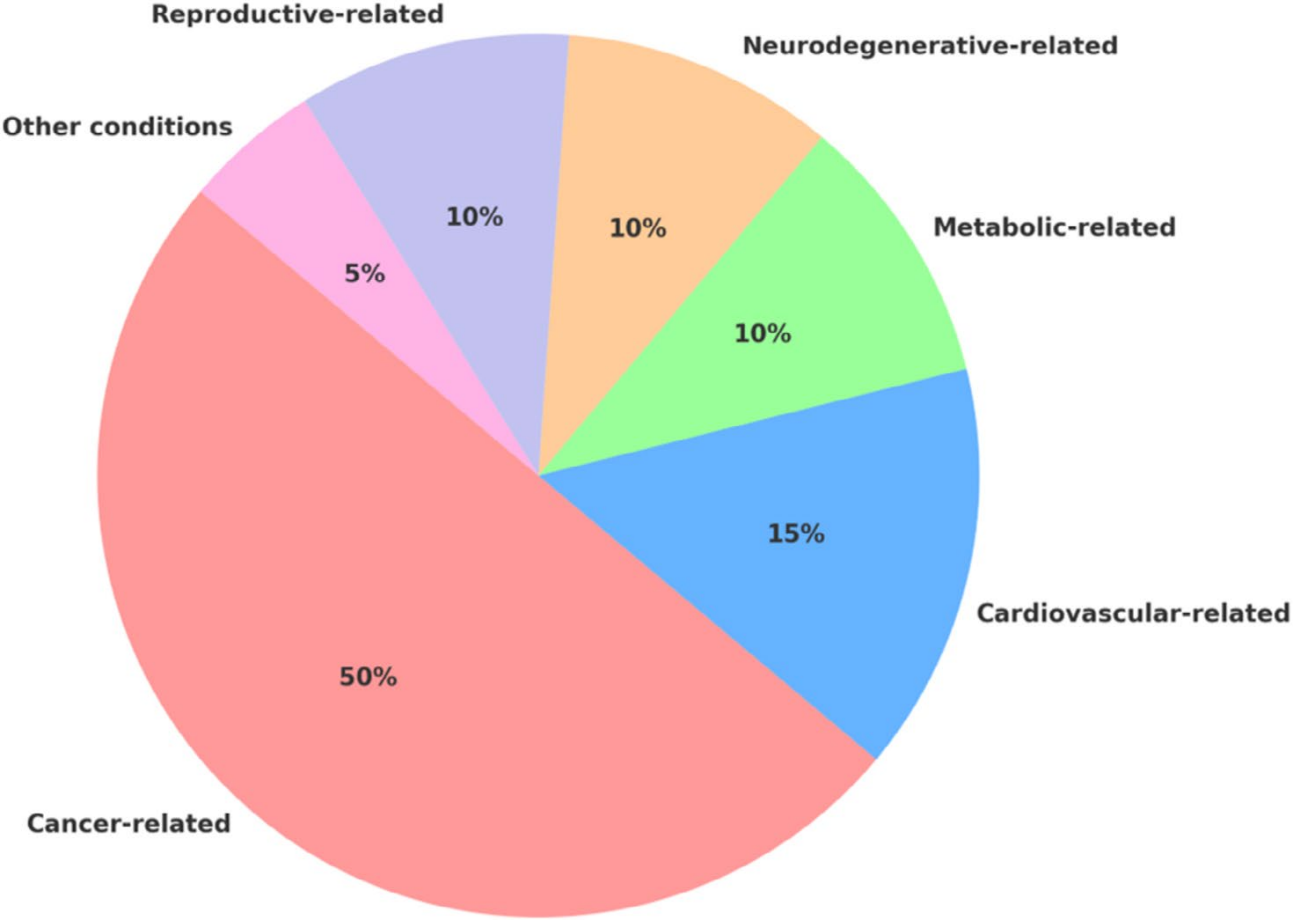
Disease distribution of antioxidant–circRNA studies. Pie chart illustrating the distribution of disease areas addressed by the reviewed antioxidant–circRNA studies. *Cancer‐related* studies constitute the largest segment (~50%), underscoring that many natural antioxidants (e.g., quercetin, curcumin, genistein) have been investigated for circRNA‐mediated anticancer effects. *Cardiovascular‐related* contexts (e.g., atherosclerosis, cardiac hypertrophy) account for ~15%, whereas *metabolic* conditions (diabetes, obesity) ~10%. *Neurodegenerative* disease models (e.g., antioxidant effects in Parkinson's or Alzheimer's via circRNAs) comprise ~10%, and *reproductive*/developmental studies (fertility, embryonic development) another ~10%. Other inflammatory or injury models (~5%) make up the remainder. This distribution highlights that cancer is the predominant focus of current antioxidant–circRNA research, followed by cardiovascular and metabolic diseases.

Each class of antioxidants—flavonoids, carotenoids, polyphenols, vitamins, terpenoids, and alkaloids—demonstrates distinct structural and biochemical features that influence specific circRNA‐mediated signaling pathways. Notably, compounds such as quercetin, curcumin, resveratrol, astaxanthin, and genistein have consistently demonstrated the ability to regulate circRNAs implicated in cancer, cardiovascular disease, neurodegeneration, and metabolic dysfunction (Figure 7). These findings highlight the dual function of antioxidants not only as scavengers of ROS but also as potent regulators of ncRNA biology.

The therapeutic potential of targeting circRNA pathways using natural antioxidants lies in their ability to correct dysregulated gene expression with high specificity and relatively low toxicity. Nonetheless, key challenges must be addressed: we need a better mechanistic understanding of antioxidant–circRNA interactions, more robust methods for detecting and functionally testing circRNA in vivo, and improved delivery of antioxidants (pharmacokinetics) to target tissues. Emerging strategies, such as nanoparticle‐based formulations and combinatorial therapies integrating RNA‐targeting tools, may accelerate the clinical translation of these approaches.

In summary, natural antioxidants emerge as promising modulators of circRNA regulatory circuits, offering a new dimension to redox biology and RNA‐based therapeutics. Continued interdisciplinary efforts integrating molecular biology, pharmacology, bioinformatics, and clinical science will be essential to unlock the full potential of antioxidant‐driven circRNA modulation for disease prevention and treatment; ultimately paving the way for novel RNA‐based therapies in the clinic.

## Author Contributions

**Aseela Fathima:** visualization (equal), writing – original draft (equal). **Shadiya Fawzu Ameer:** visualization (equal), writing – original draft (equal). **Rabia Ilhem Kerzabi:** visualization (equal), writing – original draft (equal). **Roberta Giordo:** visualization (equal), writing – review and editing (equal). **Gheyath K. Nasrallah:** visualization (equal), writing – review and editing (equal). **Hatem Zayed:** project administration (equal), resources (lead), writing – review and editing (equal). **Gianfranco Pintus:** conceptualization (lead), funding acquisition (lead), project administration (lead), visualization (equal), writing – review and editing (lead).

## Ethics Statement

The authors have nothing to report.

## Consent

The authors have nothing to report.

## Conflicts of Interest

The authors declare no conflicts of interest.

## Related WIREs Articles

Regulation of microRNA by circular RNA

## Data Availability

Data sharing is not applicable to this article as no new data were created or analyzed in this study.

## References

[wrna70023-bib-0001] Abdulaal, W. H. , H. Z. Asfour , N. Helmi , et al. 2024. “Capsaicin Ameliorate Pulmonary Fibrosis via Antioxidant Nrf‐2/ PPAR‐ γ Pathway Activation and Inflammatory TGF‐β1/ NF‐κB/COX II Pathway Inhibition.” Frontiers in Pharmacology 15: 1333715. 10.3389/fphar.2024.1333715. 38449809 PMC10915016

[wrna70023-bib-0002] Akhter, R. 2018. “Circular RNA and Alzheimer's Disease.” In Circular RNAs, edited by J. Xiao , vol. 1087, 239–243. Springer. 10.1007/978-981-13-1426-1_19. 30259371

[wrna70023-bib-0003] Albadrani, G. M. , M. N. BinMowyna , M. N. Bin‐Jumah , G. El–Akabawy , H. Aldera , and A. M. AL‐Farga . 2021. “Quercetin Prevents Myocardial Infarction Adverse Remodeling in Rats by Attenuating TGF‐β1/Smad3 Signaling: Different Mechanisms of Action.” Saudi Journal of Biological Sciences 28, no. 5: 2772–2782. 10.1016/j.sjbs.2021.02.007. 34012318 PMC8116976

[wrna70023-bib-0004] Ali, M. , M. Hassan , S. A. Ansari , H. M. Alkahtani , L. S. Al‐Rasheed , and S. A. Ansari . 2024. “Quercetin and Kaempferol as Multi‐Targeting Antidiabetic Agents Against Mouse Model of Chemically Induced Type 2 Diabetes.” Pharmaceuticals 17, no. 6: 6. 10.3390/ph17060757. PMC11206732 38931424

[wrna70023-bib-0005] Ambati, R. R. , S.‐M. Phang , S. Ravi , and R. G. Aswathanarayana . 2014. “Astaxanthin: Sources, Extraction, Stability, Biological Activities and Its Commercial Applications—A Review.” Marine Drugs 12, no. 1: 1. 10.3390/md12010128. PMC3917265 24402174

[wrna70023-bib-0006] An, P. , X. Li , Y. Zhao , et al. 2025. “Curcumin Alleviates Renal Fibrosis in Chronic Kidney Disease by Targeting the circ_0008925‐Related Pathway.” Renal Failure 47, no. 1: 2444393. 10.1080/0886022X.2024.2444393. 40038566 PMC11884099

[wrna70023-bib-0007] Anand David, A. V. , R. Arulmoli , and S. Parasuraman . 2016. “Overviews of Biological Importance of Quercetin: A Bioactive Flavonoid.” Pharmacognosy Reviews 10, no. 20: 84–89. 10.4103/0973-7847.194044. 28082789 PMC5214562

[wrna70023-bib-0008] Aung, T. N. , Z. Qu , R. D. Kortschak , and D. L. Adelson . 2017. “Understanding the Effectiveness of Natural Compound Mixtures in Cancer Through Their Molecular Mode of Action.” International Journal of Molecular Sciences 18, no. 3: 3. 10.3390/ijms18030656. 28304343 PMC5372668

[wrna70023-bib-0009] Aziz, N. , M.‐Y. Kim , and J. Y. Cho . 2018. “Anti‐Inflammatory Effects of Luteolin: A Review of In Vitro, In Vivo, and In Silico Studies.” Journal of Ethnopharmacology 225: 342–358. 10.1016/j.jep.2018.05.019. 29801717

[wrna70023-bib-0010] Babin, L. , E. Andraos , S. Fuchs , S. Pyronnet , E. Brunet , and F. Meggetto . 2021. “From circRNAs to Fusion circRNAs in Hematological Malignancies.” JCI Insight 6, no. 21: e151513. 10.1172/jci.insight.151513. 34747369 PMC8663548

[wrna70023-bib-0011] Baralic, I. , M. Andjelkovic , B. Djordjevic , et al. 2015. “Effect of Astaxanthin Supplementation on Salivary IgA, Oxidative Stress, and Inflammation in Young Soccer Players.” Evidence‐Based Complementary and Alternative Medicine 2015, no. 1: 783761. 10.1155/2015/783761. 26167194 PMC4488551

[wrna70023-bib-0012] Barbagallo, D. , A. Caponnetto , M. Cirnigliaro , et al. 2018. “CircSMARCA5 Inhibits Migration of Glioblastoma Multiforme Cells by Regulating a Molecular Axis Involving Splicing Factors SRSF1/SRSF3/PTB.” International Journal of Molecular Sciences 19, no. 2: 480. 10.3390/ijms19020480. 29415469 PMC5855702

[wrna70023-bib-0013] Bian, Y. , J. Lei , J. Zhong , et al. 2022. “Kaempferol Reduces Obesity, Prevents Intestinal Inflammation, and Modulates Gut Microbiota in High‐Fat Diet Mice.” Journal of Nutritional Biochemistry 99: 108840. 10.1016/j.jnutbio.2021.108840. 34419569

[wrna70023-bib-0014] Boin, F. , G. L. Erre , A. M. Posadino , et al. 2014. “Oxidative Stress‐Dependent Activation of Collagen Synthesis Is Induced in Human Pulmonary Smooth Muscle Cells by Sera From Patients With Scleroderma‐Associated Pulmonary Hypertension.” Orphanet Journal of Rare Diseases 9, no. 1: 123. 10.1186/s13023-014-0123-7. 25085432 PMC4237898

[wrna70023-bib-0015] Carr, A. , and S. Maggini . 2017. “Vitamin C and Immune Function.” Nutrients 9, no. 11: 1211. 10.3390/nu9111211. 29099763 PMC5707683

[wrna70023-bib-0016] Chai, Y. , J. Xu , and B. Yan . 2017. “The Anti‐Metastatic Effect of Baicalein on Colorectal Cancer.” Oncology Reports 37, no. 4: 2317–2323. 10.3892/or.2017.5437. 28259937

[wrna70023-bib-0017] Chang, W. 2017. “Non‐Coding RNAs and Berberine: A New Mechanism of Its Anti‐Diabetic Activities.” European Journal of Pharmacology 795: 8–12. 10.1016/j.ejphar.2016.11.055. 27915042

[wrna70023-bib-0018] Chaudhary, R. , B. R. Muys , I. Grammatikakis , et al. 2020. “A Circular RNA From the *MDM2* Locus Controls Cell Cycle Progression by Suppressing p53 Levels.” Molecular and Cellular Biology 40, no. 9: e00473‐19. 10.1128/MCB.00473-19. 32041821 PMC7156218

[wrna70023-bib-0019] Che, J. , B. Liang , Y. Zhang , Y. Wang , J. Tang , and G. Shi . 2017. “Kaempferol Alleviates Ox‐LDL‐Induced Apoptosis by Up‐Regulation of Autophagy via Inhibiting PI3K/Akt/mTOR Pathway in Human Endothelial Cells.” Cardiovascular Pathology 31: 57–62. 10.1016/j.carpath.2017.08.001. 28985493

[wrna70023-bib-0020] Chen, C. , H. Tan , J. Bi , et al. 2018. “Identification of Competing Endogenous RNA Regulatory Networks in Vitamin A Deficiency‐Induced Congenital Scoliosis by Transcriptome Sequencing Analysis.” Cellular Physiology and Biochemistry 48, no. 5: 2134–2146. 10.1159/000492556. 30110682

[wrna70023-bib-0021] Chen, C. , D. Zhu , S. Zhang , and W. Zhang . 2022. “Identification of circRNA/miRNA/mRNA Regulatory Network Involving (+)‐Catechin Ameliorates Diabetic Nephropathy Mice.” Food Science and Human Wellness 11, no. 3: 660–668. 10.1016/j.fshw.2021.12.023.

[wrna70023-bib-0022] Chen, D. , F.‐J. Chou , Y. Chen , et al. 2022. “Targeting the Radiation‐Induced ARv7‐Mediated circNHS/miR‐512‐5p/XRCC5 Signaling With Quercetin Increases Prostate Cancer Radiosensitivity.” Journal of Experimental & Clinical Cancer Research 41, no. 1: 235. 10.1186/s13046-022-02287-4. 35918767 PMC9347162

[wrna70023-bib-0023] Chen, H. , H. Yao , J. Chi , et al. 2023. “Engineered Exosomes as Drug and RNA Co‐Delivery System: New Hope for Enhanced Therapeutics?” Frontiers in Bioengineering and Biotechnology 11: 1254356. 10.3389/fbioe.2023.1254356. 37823027 PMC10562639

[wrna70023-bib-0024] Chen, L. , and J. S. Khillan . 2010. “A Novel Signaling by Vitamin A/Retinol Promotes Self Renewal of Mouse Embryonic Stem Cells by Activating PI3K/Akt Signaling Pathway via Insulin‐Like Growth Factor‐1 Receptor.” Stem Cells 28, no. 1: 57–63. 10.1002/stem.251. 19890980

[wrna70023-bib-0025] Chen, Q. , H. Guo , Y. Zong , and X. Zhao . 2022. “Curcumin Restrains Hepatocellular Carcinoma Progression Depending on the Regulation of the circ_0078710/miR‐378b/PRIM2 Axis.” Journal of Receptors and Signal Transduction 42, no. 3: 313–324. 10.1080/10799893.2021.1936554. 34139933

[wrna70023-bib-0026] Chen, S. , R. F. Thorne , X. D. Zhang , M. Wu , and L. Liu . 2021. “Non‐Coding RNAs, Guardians of the p53 Galaxy.” Seminars in Cancer Biology 75: 72–83. 10.1016/j.semcancer.2020.09.002. 32927018

[wrna70023-bib-0027] Chen, X. , and Y. Lu . 2021. “Circular RNA: Biosynthesis In Vitro.” Frontiers in Bioengineering and Biotechnology 9: 787881. 10.3389/fbioe.2021.787881. 34917603 PMC8670002

[wrna70023-bib-0028] Cheng, C.‐S. , J. Chen , H.‐Y. Tan , N. Wang , Z. Chen , and Y. Feng . 2018. “ *Scutellaria baicalensis* and Cancer Treatment: Recent Progress and Perspectives in Biomedical and Clinical Studies.” American Journal of Chinese Medicine 46, no. 1: 25–54. 10.1142/S0192415X18500027. 29316796

[wrna70023-bib-0029] Chintong, S. , W. Phatvej , U. Rerk‐Am , Y. Waiprib , and W. Klaypradit . 2019. “In Vitro Antioxidant, Antityrosinase, and Cytotoxic Activities of Astaxanthin From Shrimp Waste.” Antioxidants 8, no. 5: 5. 10.3390/antiox8050128. PMC6562539 31085994

[wrna70023-bib-0030] Choi, J.‐H. , S.‐E. Park , S.‐J. Kim , and S. Kim . 2015. “Kaempferol Inhibits Thrombosis and Platelet Activation.” Biochimie 115: 177–186. 10.1016/j.biochi.2015.06.001. 26073152

[wrna70023-bib-0031] Cicero, A. F. G. , and E. Tartagni . 2012. “Antidiabetic Properties of Berberine: From Cellular Pharmacology to Clinical Effects.” Hospital Practice 40, no. 2: 56–63. 10.3810/hp.2012.04.970. 22615079

[wrna70023-bib-0032] Conn, S. J. , K. A. Pillman , J. Toubia , et al. 2015. “The RNA Binding Protein Quaking Regulates Formation of circRNAs.” Cell 160, no. 6: 1125–1134. 10.1016/j.cell.2015.02.014. 25768908

[wrna70023-bib-0033] Cui, L. , L. Feng , Z. H. Zhang , and X. B. Jia . 2014. “The Anti‐Inflammation Effect of Baicalin on Experimental Colitis Through Inhibiting TLR4/NF‐κB Pathway Activation.” International Immunopharmacology 23, no. 1: 294–303. 10.1016/j.intimp.2014.09.005. 25239813

[wrna70023-bib-0034] Cui, Y. , J. Fan , W. Shi , and Z. Zhou . 2022. “Circ_0001667 Knockdown Blocks Cancer Progression and Attenuates Adriamycin Resistance by Depleting ncoa3 via Releasing mir−4458 in Breast Cancer.” Drug Development Research 83, no. 1: 75–87. 10.1002/ddr.21845. 34227151

[wrna70023-bib-0035] Da, J. , M. Xu , Y. Wang , W. Li , M. Lu , and Z. Wang . 2019. “Kaempferol Promotes Apoptosis While Inhibiting Cell Proliferation via Androgen‐Dependent Pathway and Suppressing Vasculogenic Mimicry and Invasion in Prostate Cancer.” Analytical Cellular Pathology 2019, no. 1: 1907698. 10.1155/2019/1907698. PMC6913338 31871879

[wrna70023-bib-0036] Dang, Q. , W. Song , D. Xu , et al. 2015. “Kaempferol Suppresses Bladder Cancer Tumor Growth by Inhibiting Cell Proliferation and Inducing Apoptosis.” Molecular Carcinogenesis 54, no. 9: 831–840. 10.1002/mc.22154. 24700700

[wrna70023-bib-0037] Devi, K. P. , D. S. Malar , S. F. Nabavi , et al. 2015. “Kaempferol and Inflammation: From Chemistry to Medicine.” Pharmacological Research 99: 1–10. 10.1016/j.phrs.2015.05.002. 25982933

[wrna70023-bib-0038] Digby, B. , S. Finn , and P. Ó Broin . 2024. “Computational Approaches and Challenges in the Analysis of circRNA Data.” BMC Genomics 25, no. 1: 527. 10.1186/s12864-024-10420-0. 38807085 PMC11134749

[wrna70023-bib-0039] Dogan, M. 2022. “Assessment of Mechanism Involved in the Apoptotic and Anti‐Cancer Activity of Quercetin and Quercetin‐Loaded Chitosan Nanoparticles.” Medical Oncology 39, no. 11: 176. 10.1007/s12032-022-01820-x. 35999475

[wrna70023-bib-0040] Dong, T. , N. M. Matos Pires , Z. Yang , et al. 2025. “Advances in Molecular Assays and Biosensors for Circular RNA‐Based Diagnostics and Therapeutic Monitoring.” TrAC Trends in Analytical Chemistry 183: 118112. 10.1016/j.trac.2024.118112.

[wrna70023-bib-0041] Drula, R. , C. Braicu , and I. Berindan‐Neagoe . 2024. “Current Advances in Circular RNA Detection and Investigation Methods: Are We Running in Circles?” WIREs RNA 15, no. 3: e1850. 10.1002/wrna.1850. 38702943

[wrna70023-bib-0042] Du, W. W. , W. Yang , X. Li , et al. 2018. “A Circular RNA Circ‐DNMT1 Enhances Breast Cancer Progression by Activating Autophagy.” Oncogene 37, no. 44: 5829–5842. 10.1038/s41388-018-0369-y. 29973691

[wrna70023-bib-0043] Du, W. W. , W. Yang , E. Liu , Z. Yang , P. Dhaliwal , and B. B. Yang . 2016. “Foxo3 Circular RNA Retards Cell Cycle Progression via Forming Ternary Complexes With p21 and CDK2.” Nucleic Acids Research 44, no. 6: 2846–2858. 10.1093/nar/gkw027. 26861625 PMC4824104

[wrna70023-bib-0044] Ensoy, M. , Z. S. Bumin , H. A. Jama , and D. Cansaran‐Duman . 2023. “The Regulation Role of Ferroptosis Mechanism of Anti‐Cancer Drugsand Noncoding RNAs.” Current Medicinal Chemistry 30, no. 14: 1638–1656. 10.2174/0929867329666220629154418. 35770401

[wrna70023-bib-0045] Fakhri, S. , F. Abbaszadeh , L. Dargahi , and M. Jorjani . 2018. “Astaxanthin: A Mechanistic Review on Its Biological Activities and Health Benefits.” Pharmacological Research 136: 1–20. 10.1016/j.phrs.2018.08.012. 30121358

[wrna70023-bib-0046] Fan, Y. , J. Wang , W. Jin , et al. 2021. “CircNR3C2 Promotes HRD1‐Mediated Tumor‐Suppressive Effect via Sponging miR‐513a‐3p in Triple‐Negative Breast Cancer.” Molecular Cancer 20, no. 1: 25. 10.1186/s12943-021-01321-x. 33530981 PMC7851937

[wrna70023-bib-0047] Fang, L. , W. W. Du , J. Lyu , et al. 2018. “Enhanced Breast Cancer Progression by Mutant p53 Is Inhibited by the Circular RNA Circ‐Ccnb1.” Cell Death and Differentiation 25, no. 12: 2195–2208. 10.1038/s41418-018-0115-6. 29795334 PMC6261950

[wrna70023-bib-0048] Farazi, M. M. , F. Rostamzadeh , S. Jafarinejad‐Farsangi , M. Moazam Jazi , E. Jafari , and S. Gharbi . 2024. “CircPAN3/miR‐221/PTEN Axis and Apoptosis in Myocardial Infarction: Quercetin's Regulatory Effects.” Gene 909: 148316. 10.1016/j.gene.2024.148316. 38401834

[wrna70023-bib-0049] Farruggia, C. , M.‐B. Kim , M. Bae , et al. 2018. “Astaxanthin Exerts Anti‐Inflammatory and Antioxidant Effects in Macrophages in NRF2‐Dependent and Independent Manners.” Journal of Nutritional Biochemistry 62: 202–209. 10.1016/j.jnutbio.2018.09.005. 30308382

[wrna70023-bib-0050] Feng, D. , Z. Li , G. Wang , et al. 2018. “Microarray Analysis of Differentially Expressed Profiles of Circular RNAs in a Mouse Model of Intestinal Ischemia/Reperfusion Injury With and Without Ischemic Postconditioning.” Cellular Physiology and Biochemistry 48, no. 4: 1579–1594. 10.1159/000492280. 30071511

[wrna70023-bib-0051] Flieger, J. , M. Raszewska‐Famielec , E. Radzikowska‐Büchner , and W. Flieger . 2024. “Skin Protection by Carotenoid Pigments.” International Journal of Molecular Sciences 25, no. 3: 1431. 10.3390/ijms25031431. 38338710 PMC10855854

[wrna70023-bib-0052] Fois, A. G. , A. M. Posadino , R. Giordo , et al. 2018. “Antioxidant Activity Mediates Pirfenidone Antifibrotic Effects in Human Pulmonary Vascular Smooth Muscle Cells Exposed to Sera of Idiopathic Pulmonary Fibrosis Patients.” Oxidative Medicine and Cellular Longevity 2018, no. 1: 2639081. 10.1155/2018/2639081. 30420906 PMC6215550

[wrna70023-bib-0053] Garg, M. , S. K. Chaudhary , A. Goyal , et al. 2022. “Comprehensive Review on Therapeutic and Phytochemical Exploration of Diosmetin: A Promising Moiety.” Phytomedicine Plus 2, no. 1: 100179. 10.1016/j.phyplu.2021.100179.

[wrna70023-bib-0054] Geng, X. , Y. Jia , Y. Zhang , et al. 2020. “Circular RNA: Biogenesis, Degradation, Functions and Potential Roles in Mediating Resistance to Anticarcinogens.” Epigenomics 12, no. 3: 267–283. 10.2217/epi-2019-0295. 31808351

[wrna70023-bib-0055] Giordo, R. , F. A. M. Ahmadi , N. A. Husaini , et al. 2024. “microRNA 21 and Long Non‐Coding RNAs Interplays Underlie Cancer Pathophysiology: A Narrative Review.” Non‐Coding RNA Research 9, no. 3: 831–852. 10.1016/j.ncrna.2024.03.013. 38586315 PMC10995982

[wrna70023-bib-0056] Giordo, R. , Y. M. A. Ahmed , H. Allam , et al. 2021. “EndMT Regulation by Small RNAs in Diabetes‐Associated Fibrotic Conditions: Potential Link With Oxidative Stress.” Frontiers in Cell and Developmental Biology 9: 683594. 10.3389/fcell.2021.683594. 34095153 PMC8170089

[wrna70023-bib-0057] Giordo, R. , A. Cossu , V. Pasciu , P. T. Hoa , A. M. Posadino , and G. Pintus . 2013. “Different Redox Response Elicited by Naturally Occurring Antioxidants in Human Endothelial Cells.” Open Biochemistry Journal 7: 44–53. 10.2174/1874091X01307010044. 23730364 PMC3664460

[wrna70023-bib-0058] Giordo, R. , A. Cossu , M. C. Porcu , et al. 2023. “Cytoprotective, Antioxidant, and Anti‐Migratory Activity of * Pistacia lentiscus L*. Supercritical Carbon Dioxide Extract on Primary Human Endothelial Cells.” Natural Product Research 37, no. 16: 2681–2687. 10.1080/14786419.2022.2130304. 36200704

[wrna70023-bib-0059] Giordo, R. , Z. Wehbe , A. M. Posadino , et al. 2022. “Disease‐Associated Regulation of Non‐Coding RNAs by Resveratrol: Molecular Insights and Therapeutic Applications.” Frontiers in Cell and Developmental Biology 10: 894305. 10.3389/fcell.2022.894305. 35912113 PMC9326031

[wrna70023-bib-0060] Glaviano, A. , A. S. C. Foo , H. Y. Lam , et al. 2023. “PI3K/AKT/mTOR Signaling Transduction Pathway and Targeted Therapies in Cancer.” Molecular Cancer 22, no. 1: 138. 10.1186/s12943-023-01827-6. 37596643 PMC10436543

[wrna70023-bib-0061] Goetz, R. , and M. Mohammadi . 2013. “Exploring Mechanisms of FGF Signalling Through the Lens of Structural Biology.” Nature Reviews Molecular Cell Biology 14, no. 3: 166–180. 10.1038/nrm3528. 23403721 PMC3695728

[wrna70023-bib-0062] Goina, C. A. , D. M. Goina , S. S. Farcas , and N. I. Andreescu . 2024. “The Role of Circular RNA for Early Diagnosis and Improved Management of Patients With Cardiovascular Diseases.” International Journal of Molecular Sciences 25, no. 5: 5. 10.3390/ijms25052986. PMC10932049 38474233

[wrna70023-bib-0063] Greene, J. , A.‐M. Baird , L. Brady , et al. 2017. “Circular RNAs: Biogenesis, Function and Role in Human Diseases.” Frontiers in Molecular Biosciences 4: 38. 10.3389/fmolb.2017.00038. 28634583 PMC5459888

[wrna70023-bib-0064] Guo, Z. , X. Hu , Z. Xing , et al. 2015. “Baicalein Inhibits Prostate Cancer Cell Growth and Metastasis via the Caveolin‐1/AKT/mTOR Pathway.” Molecular and Cellular Biochemistry 406, no. 1: 111–119. 10.1007/s11010-015-2429-8. 25957503 PMC4502300

[wrna70023-bib-0065] Hacioglu, C. 2022. “Capsaicin Inhibits Cell Proliferation by Enhancing Oxidative Stress and Apoptosis Through SIRT1/NOX4 Signaling Pathways in HepG2 and HL‐7702 Cells.” Journal of Biochemical and Molecular Toxicology 36, no. 3: e22974. 10.1002/jbt.22974. 34939720

[wrna70023-bib-0066] Hansen, T. B. , E. D. Wiklund , J. B. Bramsen , et al. 2011. “miRNA‐Dependent Gene Silencing Involving Ago2‐Mediated Cleavage of a Circular Antisense RNA: miRNA Mediated Cleavage of Circular Antisense RNA.” EMBO Journal 30, no. 21: 4414–4422. 10.1038/emboj.2011.359. 21964070 PMC3230379

[wrna70023-bib-0067] Hao, R. , F. Li , X. Song , X. Tan , D. Sun‐Waterhouse , and D. Li . 2020. “Caffeic Acid Phenethyl Ester Against Cadmium Induced Toxicity Mediated by CircRNA Modulates Autophagy in HepG2 Cells.” Ecotoxicology and Environmental Safety 197: 110610. 10.1016/j.ecoenv.2020.110610. 32298858

[wrna70023-bib-0068] Hayakawa, S. , T. Ohishi , Y. Oishi , M. Isemura , and N. Miyoshi . 2022. “Contribution of Non‐Coding RNAs to Anticancer Effects of Dietary Polyphenols: Chlorogenic Acid, Curcumin, Epigallocatechin‐3‐Gallate, Genistein, Quercetin and Resveratrol.” Antioxidants 11, no. 12: 2352. 10.3390/antiox11122352. 36552560 PMC9774417

[wrna70023-bib-0069] He, J. , H. Fu , C. Li , Z. Deng , and H. Chang . 2022. “Eriodictyol Inhibits Breast Carcinogenesis by Targeting circ_0007503 and Repressing PI3K/Akt Pathway.” Phytomedicine 102: 154159. 10.1016/j.phymed.2022.154159. 35580441

[wrna70023-bib-0070] Hossain, R. , C. Quispe , R. A. Khan , et al. 2022. “Propolis: An Update on Its Chemistry and Pharmacological Applications.” Chinese Medicine 17, no. 1: 100. 10.1186/s13020-022-00651-2. 36028892 PMC9412804

[wrna70023-bib-0071] Hu, J. , R. Wang , Y. Liu , J. Zhou , K. Shen , and Y. Dai . 2021. “Baicalein Represses Cervical Cancer Cell Growth, Cell Cycle Progression and Promotes Apoptosis via Blocking AKT/mTOR Pathway by the Regulation of circHIAT1/miR‐19a‐3p Axis.” Oncotargets and Therapy 14: 905–916. 10.2147/OTT.S282790. 33603395 PMC7881781

[wrna70023-bib-0072] Hu, Y. , L. Cheng , S. Du , K. Wang , and S. Liu . 2024. “Antioxidant Curcumin Induces Oxidative Stress to Kill Tumor Cells (Review).” Oncology Letters 27, no. 2: 1–12. 10.3892/ol.2023.14200. 38192657 PMC10773205

[wrna70023-bib-0073] Huang, P. , H. Deng , C. Wang , Y. Zhou , and X. Chen . 2024. “Cellular Trafficking of Nanotechnology‐Mediated mRNA Delivery.” Advanced Materials 36, no. 13: 2307822. 10.1002/adma.202307822. 37929780

[wrna70023-bib-0074] Ikeda, Y. , S. Morikawa , M. Nakashima , et al. 2023. “CircRNAs and RNA‐Binding Proteins Involved in the Pathogenesis of Cancers or Central Nervous System Disorders.” Non‐Coding RNA 9, no. 2: 23. 10.3390/ncrna9020023. 37104005 PMC10142617

[wrna70023-bib-0075] Ikram, M. , T. J. Park , T. Ali , and M. O. Kim . 2020. “Antioxidant and Neuroprotective Effects of Caffeine Against Alzheimer's and Parkinson's Disease: Insight Into the Role of Nrf‐2 and A2AR Signaling.” Antioxidants 9, no. 9: 902. 10.3390/antiox9090902. 32971922 PMC7554764

[wrna70023-bib-0076] Imran, M. , A. Rauf , T. Abu‐Izneid , et al. 2019. “Luteolin, a Flavonoid, as an Anticancer Agent: A Review.” Biomedicine & Pharmacotherapy 112: 108612. 10.1016/j.biopha.2019.108612. 30798142

[wrna70023-bib-0077] Jamali, B. , M. Entezari , N. Babaei , and M. Hashemi . 2022. “The Evaluation of Vitamin E and TiO2 Nanoparticles Administration in Parkinson's Rat Model.” Cell Journal (Yakhteh) 25: 102. 10.22074/cellj.2022.557558.1071. PMC9968372 36840456

[wrna70023-bib-0078] Jiang, M.‐P. , W.‐X. Xu , J.‐C. Hou , Q. Xu , D.‐D. Wang , and J.‐H. Tang . 2021. “The Emerging Role of the Interactions Between Circular RNAs and RNA‐Binding Proteins in Common Human Cancers.” Journal of Cancer 12, no. 17: 5206–5219. 10.7150/jca.58182. 34335937 PMC8317540

[wrna70023-bib-0079] Joshi, P. , S. Joshi , D. Semwal , et al. 2021. “Curcumin: An Insight Into Molecular Pathways Involved in Anticancer Activity.” Mini Reviews in Medicinal Chemistry 21, no. 17: 2420–2457. 10.2174/1389557521666210122153823. 33480345

[wrna70023-bib-0080] Kadioglu, O. , J. Nass , M. E. M. Saeed , B. Schuler , and T. Efferth . 2015. Kaempferol Is an Anti‐Inflammatory Compound With Activity Towards NF‐κB Pathway Proteins. Anticancer Research. 25964540

[wrna70023-bib-0081] Kattoor, A. J. , N. V. K. Pothineni , D. Palagiri , and J. L. Mehta . 2017. “Oxidative Stress in Atherosclerosis.” Current Atherosclerosis Reports 19, no. 11: 42. 10.1007/s11883-017-0678-6. 28921056

[wrna70023-bib-0082] Kaźmierczak‐Barańska, J. , K. Boguszewska , A. Adamus‐Grabicka , and B. T. Karwowski . 2020. “Two Faces of Vitamin C—Antioxidative and Pro‐Oxidative Agent.” Nutrients 12, no. 5: 1501. 10.3390/nu12051501. 32455696 PMC7285147

[wrna70023-bib-0083] Khillan, J. 2014. “Vitamin A/Retinol and Maintenance of Pluripotency of Stem Cells.” Nutrients 6, no. 3: 1209–1222. 10.3390/nu6031209. 24662164 PMC3967188

[wrna70023-bib-0084] Khosravi, F. , V. Hojati , S. Mirzaei , M. Hashemi , and M. Entezari . 2023. “Curcumin Neuroprotective Effects in Parkinson Disease During Pregnancy.” Brain Research Bulletin 201: 110726. 10.1016/j.brainresbull.2023.110726. 37543296

[wrna70023-bib-0216] Koch, M. R. , J. B. Demeter , M. W. Shilling , et al. 2025. “The Circular RNA Landscape of Human Dorsal Root Ganglia and Its Association With Opioid Exposure.” BioRxiv. 10.1101/2025.02.26.640459.

[wrna70023-bib-0085] Kouhestani, S. , A. Jafari , and P. Babaei . 2018. “Kaempferol Attenuates Cognitive Deficit via Regulating Oxidative Stress and Neuroinflammation in an Ovariectomized Rat Model of Sporadic Dementia.” Neural Regeneration Research 13, no. 10: 1827. 10.4103/1673-5374.238714. 30136699 PMC6128063

[wrna70023-bib-0086] Kurniawan, R. , F. Nurkolis , N. A. Taslim , et al. 2023. “Carotenoids Composition of Green Algae Caulerpa Racemosa and Their Antidiabetic, Anti‐Obesity, Antioxidant, and Anti‐Inflammatory Properties.” Molecules 28, no. 7: 3267. 10.3390/molecules28073267. 37050034 PMC10096636

[wrna70023-bib-0087] Lee, Y.‐C. , H.‐E. Tzeng , H.‐H. Lin , and K.‐Y. Hsiao . 2023. “Circular RNA in Liquid Biopsy as Biomarkers Toward Precision Medicine.” Molecular Therapy—Nucleic Acids 31: 689–690. 10.1016/j.omtn.2023.02.023. 36910713 PMC9999159

[wrna70023-bib-0088] Li, F. , T.‐Y. Long , S.‐S. Bi , S. A. Sheikh , and C.‐L. Zhang . 2020. “circPAN3 Exerts a Profibrotic Role via Sponging miR‐221 Through FoxO3/ATG7‐Activated Autophagy in a Rat Model of Myocardial Infarction.” Life Sciences 257: 118015. 10.1016/j.lfs.2020.118015. 32629000

[wrna70023-bib-0089] Li, H. , Y. Wei , X. Li , et al. 2022. “Diosmetin Has Therapeutic Efficacy in Colitis Regulating Gut Microbiota, Inflammation, and Oxidative Stress via the Circ‐Sirt1/Sirt1 Axis.” Acta Pharmacologica Sinica 43, no. 4: 919–932. 10.1038/s41401-021-00726-0. 34262136 PMC8976001

[wrna70023-bib-0090] Li, N. , and D. Jiang . 2017. “Jumonji Domain Containing 2C Promotes Cell Migration and Invasion Through Modulating CUL4A Expression in Lung Cancer.” Biomedicine & Pharmacotherapy 89: 305–315. 10.1016/j.biopha.2017.02.014. 28236704

[wrna70023-bib-0091] Li, S. , M. Hao , T. Wu , et al. 2021. “Kaempferol Alleviates Human Endothelial Cell Injury Through circNOL12/miR‐6873‐3p/FRS2 Axis.” Biomedicine & Pharmacotherapy 137: 111419. 10.1016/j.biopha.2021.111419. 33761622

[wrna70023-bib-0092] Li, S. , Y. Ma , Y. Tan , et al. 2018. “Profiling and Functional Analysis of Circular RNAs in Acute Promyelocytic Leukemia and Their Dynamic Regulation During All‐Trans Retinoic Acid Treatment.” Cell Death & Disease 9, no. 6: 651. 10.1038/s41419-018-0699-2. 29844435 PMC5973936

[wrna70023-bib-0093] Li, X. , R. Chen , X. Lei , et al. 2021. “Quercetin Regulates ERα Mediated Differentiation of BMSCs Through Circular RNA.” Gene 769: 145172. 10.1016/j.gene.2020.145172. 33065239

[wrna70023-bib-0094] Li, X. , C. Niu , G. Yi , et al. 2024. “Quercetin Inhibits the Epithelial‐Mesenchymal Transition and Reverses CDK4/6 Inhibitor Resistance in Breast Cancer by Regulating circHIAT1/miR‐19a‐3p/CADM2 Axis.” PLoS One 19, no. 7: e0305612. 10.1371/journal.pone.0305612. 38990915 PMC11239024

[wrna70023-bib-0095] Li, Y. , L. Gong , N. Qin , et al. 2021. “Comprehensive Analysis of circRNA Expression Pattern and circRNA‐miRNA‐mRNA Network in Oral Squamous Cell Carcinoma.” Oral Oncology 121: 105437. 10.1016/j.oraloncology.2021.105437. 34265729

[wrna70023-bib-0096] Lian, J. , J. Tang , H. Shi , et al. 2015. “Positive Feedback Loop of Hepatoma‐Derived Growth Factor and β‐Catenin Promotes Carcinogenesis of Colorectal Cancer.” Oncotarget 6, no. 30: 29357–29374. 26296979 10.18632/oncotarget.4982 PMC4745732

[wrna70023-bib-0097] Liang, W. , G. Liu , W. Zhou , et al. 2024. “Astaxanthin Mediated Repair of tBHP‐Induced Cellular Injury in Chondrocytes.” Redox Report 29, no. 1: 2422271. 10.1080/13510002.2024.2422271. 39495906 PMC11536701

[wrna70023-bib-0098] Liao, S. , S. O. Omage , L. Börmel , et al. 2022. “Vitamin E and Metabolic Health: Relevance of Interactions With Other Micronutrients.” Antioxidants 11, no. 9: 1785. 10.3390/antiox11091785. 36139859 PMC9495493

[wrna70023-bib-0099] Liu, B. , L. Li , G. Liu , et al. 2021. “Baicalein Attenuates Cardiac Hypertrophy in Mice via Suppressing Oxidative Stress and Activating Autophagy in Cardiomyocytes.” Acta Pharmacologica Sinica 42, no. 5: 701–714. 10.1038/s41401-020-0496-1. 32796955 PMC8115069

[wrna70023-bib-0100] Liu, J. , L. Fu , F. Yin , et al. 2023. “Diosmetin Maintains Barrier Integrity by Reducing the Expression of ABCG2 in Colonic Epithelial Cells.” Journal of Agricultural and Food Chemistry 71, no. 23: 8931–8940. 10.1021/acs.jafc.3c00912. 37269551

[wrna70023-bib-0101] Liu, J. , Y. Wei , Y. Lin , et al. 2021. “Expression of the Circular RNAs in Astaxanthin Promotes Cholesterol Efflux From THP‐1 Cells Based on RNA‐Seq.” Genes & Nutrition 16, no. 1: 13. 10.1186/s12263-021-00693-5. 34454424 PMC8403398

[wrna70023-bib-0102] Liu, K.‐S. , F. Pan , X.‐D. Mao , C. Liu , and Y.‐J. Chen . 2019. “Biological Functions of Circular RNAs and Their Roles in Occurrence of Reproduction and Gynecological Diseases.” American Journal of Translational Research 11, no. 1: 1–15. 30787966 PMC6357300

[wrna70023-bib-0103] Liu, S. , X. Y. Guo , Q. J. Shang , and P. Gao . 2023. “The Biogenesis, Biological Functions and Modification of Circular RNAs.” Experimental and Molecular Pathology 131: 104861. 10.1016/j.yexmp.2023.104861. 37156323

[wrna70023-bib-0104] Liu, X. , Y. Liu , Z. Liu , et al. 2021. “CircMYH9 Drives Colorectal Cancer Growth by Regulating Serine Metabolism and Redox Homeostasis in a p53‐Dependent Manner.” Molecular Cancer 20: 114. 10.1186/s12943-021-01412-9. 34496888 PMC8424912

[wrna70023-bib-0105] Lotfi, N. , Z. Yousefi , M. Golabi , et al. 2023. “The Potential Anti‐Cancer Effects of Quercetin on Blood, Prostate and Lung Cancers: An Update.” Frontiers in Immunology 14: 1077531. 10.3389/fimmu.2023.1077531. 36926328 PMC10011078

[wrna70023-bib-0106] Lu, J. , Y. Cao , K. Cheng , et al. 2015. “Berberine Regulates Neurite Outgrowth Through AMPK‐Dependent Pathways by Lowering Energy Status.” Experimental Cell Research 334, no. 2: 194–206. 10.1016/j.yexcr.2015.04.006. 25889370

[wrna70023-bib-0107] Luo, S. , M. Deng , Z. Xie , X. Li , G. Huang , and Z. Zhou . 2021. “Circulating Circular RNAs Profiles Associated With Type 1 Diabetes.” Diabetes/Metabolism Research and Reviews 37, no. 3: e3394. 10.1002/dmrr.3394. 32798322

[wrna70023-bib-0108] Luo, Y. , P. Shang , and D. Li . 2017. “Luteolin: A Flavonoid That Has Multiple Cardio‐Protective Effects and Its Molecular Mechanisms.” Frontiers in Pharmacology 8: 692. 10.3389/fphar.2017.00692. 29056912 PMC5635727

[wrna70023-bib-0109] M. Kaminski, B. , D. Steinhilber , J. M. Stein , and S. Ulrich . 2012. “Phytochemicals Resveratrol and Sulforaphane as Potential Agents for Enhancing the Anti‐Tumor Activities of Conventional Cancer Therapies.” Current Pharmaceutical Biotechnology 13, no. 1: 137–146. 10.2174/138920112798868746. 21466425

[wrna70023-bib-0110] Mahmoud, A. H. , N. M. Taha , M. Zakhary , and M. S. Tadros . 2019. “PTEN Gene & TNF‐Alpha in Acute Myocardial Infarction.” IJC Heart & Vasculature 23: 100366. 10.1016/j.ijcha.2019.100366. 31065586 PMC6495076

[wrna70023-bib-0111] Mangiavacchi, A. , G. Morelli , and V. Orlando . 2023. “Behind the Scenes: How RNA Orchestrates the Epigenetic Regulation of Gene Expression.” Frontiers in Cell and Developmental Biology 11: 1123975. 10.3389/fcell.2023.1123975. 36760365 PMC9905133

[wrna70023-bib-0112] Mansour, H. , H. Slika , S. A. Nasser , et al. 2024. “Flavonoids, Gut Microbiota and Cardiovascular Disease: Dynamics and Interplay.” Pharmacological Research 209: 107452. 10.1016/j.phrs.2024.107452. 39383791

[wrna70023-bib-0113] Mele, V. G. , T. Chioccarelli , R. Finamore , et al. 2023. “Antioxidants Positively Regulate Obesity Dependent circRNAs—Sperm Quality—Functional Axis.” Frontiers in Endocrinology 14: 1290971. 10.3389/fendo.2023.1290971. 38169845 PMC10758610

[wrna70023-bib-0114] Meng, G. , K. Chai , X. Li , Y. Zhu , and W. Huang . 2016. “Luteolin Exerts Pro‐Apoptotic Effect and Anti‐Migration Effects on A549 Lung Adenocarcinoma Cells Through the Activation of MEK/ERK Signaling Pathway.” Chemico‐Biological Interactions 257: 26–34. 10.1016/j.cbi.2016.07.028. 27474067

[wrna70023-bib-0115] Michalak, M. 2022. “Plant‐Derived Antioxidants: Significance in Skin Health and the Ageing Process.” International Journal of Molecular Sciences 23, no. 2: 585. 10.3390/ijms23020585. 35054770 PMC8776015

[wrna70023-bib-0116] Mondal, P. , J. Natesh , D. Penta , and S. M. Meeran . 2022. “Progress and Promises of Epigenetic Drugs and Epigenetic Diets in Cancer Prevention and Therapy: A Clinical Update.” Seminars in Cancer Biology 83: 503–522. 10.1016/j.semcancer.2020.12.006. 33309850

[wrna70023-bib-0117] Mu, J. , T. Liu , L. Jiang , et al. 2016. “The Traditional Chinese Medicine Baicalein Potently Inhibits Gastric Cancer Cells.” Journal of Cancer 7, no. 4: 453–461. 10.7150/jca.13548. 26918059 PMC4749366

[wrna70023-bib-0118] Nabavi, S. F. , N. Braidy , O. Gortzi , et al. 2015. “Luteolin as an Anti‐Inflammatory and Neuroprotective Agent: A Brief Review.” Brain Research Bulletin 119: 1–11. 10.1016/j.brainresbull.2015.09.002. 26361743

[wrna70023-bib-0119] Nielsen, A. F. , A. Bindereif , I. Bozzoni , et al. 2022. “Best Practice Standards for circRNA Research.” Nature Methods 19, no. 10: 1208–1220. 10.1038/s41592-022-01487-2. 35618955 PMC9759028

[wrna70023-bib-0120] Ntalouka, F. , and A. Tsirivakou . 2023. “Luteolin: A Promising Natural Agent in Management of Pain in Chronic Conditions.” Frontiers in Pain Research 4: 1114428. 10.3389/fpain.2023.1114428. 36937566 PMC10016360

[wrna70023-bib-0121] Padayatty, S. , and M. Levine . 2016. “Vitamin C: The Known and the Unknown and Goldilocks.” Oral Diseases 22, no. 6: 463–493. 10.1111/odi.12446. 26808119 PMC4959991

[wrna70023-bib-0122] Padmavathi, G. , and K. M. Ramkumar . 2021. “MicroRNA Mediated Regulation of the Major Redox Homeostasis Switch, Nrf2, and Its Impact on Oxidative Stress‐Induced Ischemic/Reperfusion Injury.” Archives of Biochemistry and Biophysics 698: 108725. 10.1016/j.abb.2020.108725. 33326800

[wrna70023-bib-0123] Pasciu, V. , A. M. Posadino , A. Cossu , et al. 2010. “Akt Downregulation by Flavin Oxidase–Induced ROS Generation Mediates Dose‐Dependent Endothelial Cell Damage Elicited by Natural Antioxidants.” Toxicological Sciences 114, no. 1: 101–112. 10.1093/toxsci/kfp301. 20015842

[wrna70023-bib-0124] Paskeh, M. D. A. , N. Babaei , M. Hashemi , et al. 2024. “The Protective Impact of Curcumin, Vitamin D and E Along With Manganese Oxide and Iron (III) Oxide Nanoparticles in Rats With Scrotal Hyperthermia: Role of Apoptotic Genes, miRNA and circRNA.” Journal of Trace Elements in Medicine and Biology 81: 127320. 10.1016/j.jtemb.2023.127320. 37913559

[wrna70023-bib-0125] Pisignano, G. , D. C. Michael , T. H. Visal , R. Pirlog , M. Ladomery , and G. A. Calin . 2023. “Going Circular: History, Present, and Future of circRNAs in Cancer.” Oncogene 42, no. 38: 2783–2800. 10.1038/s41388-023-02780-w. 37587333 PMC10504067

[wrna70023-bib-0126] Posadino, A. M. , A. Cossu , R. Giordo , et al. 2013. “Coumaric Acid Induces Mitochondrial Damage and Oxidative‐Mediated Cell Death of Human Endothelial Cells.” Cardiovascular Toxicology 13, no. 3: 301–306. 10.1007/s12012-013-9205-3. 23504614

[wrna70023-bib-0127] Posadino, A. M. , G. L. Erre , A. Cossu , et al. 2022. “NADPH‐Derived ROS Generation Drives Fibrosis and Endothelial‐To‐Mesenchymal Transition in Systemic Sclerosis: Potential Cross Talk With Circulating miRNAs.” Biomolecular Concepts 13, no. 1: 11–24. 10.1515/bmc-2021-0023. 35189048

[wrna70023-bib-0128] Posadino, A. M. , R. Giordo , A. Cossu , et al. 2019. “Flavin Oxidase‐Induced ROS Generation Modulates PKC Biphasic Effect of Resveratrol on Endothelial Cell Survival.” Biomolecules 9, no. 6: 209. 10.3390/biom9060209. 31151226 PMC6628153

[wrna70023-bib-0129] Posadino, A. M. , R. Giordo , G. Pintus , et al. 2023. “Medicinal and Mechanistic Overview of Artemisinin in the Treatment of Human Diseases.” Biomedicine & Pharmacotherapy 163: 114866. 10.1016/j.biopha.2023.114866. 37182516

[wrna70023-bib-0130] Posadino, A. M. , R. Giordo , I. Ramli , et al. 2023. “An Updated Overview of Cyanidins for Chemoprevention and Cancer Therapy.” Biomedicine & Pharmacotherapy 163: 114783. 10.1016/j.biopha.2023.114783. 37121149

[wrna70023-bib-0131] Posadino, A. M. , P. Maccioccu , R. Giordo , et al. 2025. “Exploring the Medicinal Potential of *Citrus limon* Var. Pompia Camarda Var. Nova: From Phytochemicals to Therapeutic Applications.” Food Bioscience 66: 106255. 10.1016/j.fbio.2025.106255.

[wrna70023-bib-0132] Posadino, A. M. , H. T. Phu , A. Cossu , et al. 2017. “Oxidative Stress‐Induced Akt Downregulation Mediates Green Tea Toxicity Towards Prostate Cancer Cells.” Toxicology In Vitro 42: 255–262. 10.1016/j.tiv.2017.05.005. 28495234

[wrna70023-bib-0133] Posadino, A. M. , M. C. Porcu , B. Marongiu , et al. 2012. “Antioxidant Activity of Supercritical Carbon Dioxide Extracts of Salvia Desoleana on Two Human Endothelial Cell Models.” Food Research International 46, no. 1: 354–359. 10.1016/j.foodres.2011.12.019.

[wrna70023-bib-0134] Pu, Y. , Y. Han , Y. Ouyang , et al. 2024. “Kaempferol Inhibits Colorectal Cancer Metastasis Through circ_0000345 Mediated JMJD2C/β‐Catenin Signalling Pathway.” Phytomedicine 128: 155261. 10.1016/j.phymed.2023.155261. 38493716

[wrna70023-bib-0135] Qi, M. , and S. Xin . 2019. “FGF Signaling Contributes to Atherosclerosis by Enhancing the Inflammatory Response in Vascular Smooth Muscle Cells.” Molecular Medicine Reports 20, no. 1: 162–170. 10.3892/mmr.2019.10249. 31115530 PMC6579995

[wrna70023-bib-0137] Ramli, I. , T. Cheriet , A. M. Posadino , et al. 2023. “Potential Therapeutic Targets of Resveratrol in the Prevention and Treatment of Pulmonary Fibrosis.” Frontiers in Bioscience‐Landmark 28, no. 9: 198. 10.31083/j.fbl2809198. 37796708

[wrna70023-bib-0136] Ramli, I. , T. Cheriet , A. M. Posadino , et al. 2024. “Modulating the p53‐MDM2 Pathway: The Therapeutic Potential of Natural Compounds in Cancer Treatment.” EXCLI Journal 23: 1397. 10.17179/EXCLI2024-7791. 39764218 PMC11701300

[wrna70023-bib-0138] Ramli, I. , T. Cheriet , D. T. B. Thuan , et al. 2024. “Potential Applications of Antofine and Its Synthetic Derivatives in Cancer Therapy: Structural and Molecular Insights.” Naunyn‐Schmiedeberg's Archives of Pharmacology 397, no. 11: 8231–8258. 10.1007/s00210-024-03180-x. 38842561

[wrna70023-bib-0139] Ramli, I. , A. M. Posadino , R. Giordo , et al. 2023. “Effect of Resveratrol on Pregnancy, Prenatal Complications and Pregnancy‐Associated Structure Alterations.” Antioxidants 12, no. 2: 341. 10.3390/antiox12020341. 36829900 PMC9952837

[wrna70023-bib-0140] Ramli, I. , A. M. Posadino , S. Zerizer , et al. 2023. “Low Concentrations of *Ambrosia maritima* L. Phenolic Extract Protect Endothelial Cells From Oxidative Cell Death Induced by H_2_O_2_ and Sera From Crohn's Disease Patients.” Journal of Ethnopharmacology 300: 115722. 10.1016/j.jep.2022.115722. 36115603

[wrna70023-bib-0141] Raverdeau, M. , and K. H. G. Mills . 2014. “Modulation of T Cell and Innate Immune Responses by Retinoic Acid.” Journal of Immunology 192, no. 7: 2953–2958. 10.4049/jimmunol.1303245. 24659788

[wrna70023-bib-0142] Ren, J. , Y. Lu , Y. Qian , B. Chen , T. Wu , and G. Ji . 2019. “Recent Progress Regarding Kaempferol for the Treatment of Various Diseases (Review).” Experimental and Therapeutic Medicine 18, no. 4: 2759–2776. 10.3892/etm.2019.7886. 31572524 PMC6755486

[wrna70023-bib-0143] Rudenko, N. N. , D. V. Vetoshkina , T. V. Marenkova , and M. M. Borisova‐Mubarakshina . 2023. “Antioxidants of Non‐Enzymatic Nature: Their Function in Higher Plant Cells and the Ways of Boosting Their Biosynthesis.” Antioxidants 12, no. 11: 11. 10.3390/antiox12112014. PMC10669185 38001867

[wrna70023-bib-0144] Sabogal‐Guáqueta, A. M. , J. I. Muñoz‐Manco , J. R. Ramírez‐Pineda , M. Lamprea‐Rodriguez , E. Osorio , and G. P. Cardona‐Gómez . 2015. “The Flavonoid Quercetin Ameliorates Alzheimer's Disease Pathology and Protects Cognitive and Emotional Function in Aged Triple Transgenic Alzheimer's Disease Model Mice.” Neuropharmacology 93: 134–145. 10.1016/j.neuropharm.2015.01.027. 25666032 PMC4387064

[wrna70023-bib-0145] Senapati, D. , V. Sharma , S. K. Rath , U. Rai , and N. Panigrahi . 2023. “Functional Implications and Therapeutic Targeting of Androgen Response Elements in Prostate Cancer.” Biochimie 214: 188–198. 10.1016/j.biochi.2023.07.012. 37460038

[wrna70023-bib-0146] Shaito, A. , M. Al‐Mansoob , S. M. S. Ahmad , et al. 2023. “Resveratrol‐Mediated Regulation of Mitochondria Biogenesis‐associatedPathways in Neurodegenerative Diseases: Molecular Insights and PotentialTherapeutic Applications.” Current Neuropharmacology 21, no. 5: 1184–1201. 10.2174/1570159X20666221012122855. 36237161 PMC10286596

[wrna70023-bib-0147] Shalkami, A. , M. Hassan , and A. Bakr . 2018. “Anti‐Inflammatory, Antioxidant and Anti‐Apoptotic Activity of Diosmin in Acetic Acid‐Induced Ulcerative Colitis.” Human & Experimental Toxicology 37, no. 1: 78–86. 10.1177/0960327117694075. 29187079

[wrna70023-bib-0148] Shang, S. , F. Hua , and Z.‐W. Hu . 2017. “The Regulation of β‐Catenin Activity and Function in Cancer: Therapeutic Opportunities.” Oncotarget 8, no. 20: 33972–33989. 10.18632/oncotarget.15687. 28430641 PMC5464927

[wrna70023-bib-0149] Sharma, U. , H. S. Tuli , V. Uttam , et al. 2022. “Role of Hedgehog and Hippo Signaling Pathways in Cancer: A Special Focus on Non‐Coding RNAs.” Pharmacological Research 186: 106523. 10.1016/j.phrs.2022.106523. 36377125

[wrna70023-bib-0150] Shu, G. , X. Lu , Y. Pan , et al. 2023. “Exosomal circSPIRE1 Mediates Glycosylation of E‐Cadherin to Suppress Metastasis of Renal Cell Carcinoma.” Oncogene 42, no. 22: 1802–1820. 10.1038/s41388-023-02678-7. 37046045 PMC10238271

[wrna70023-bib-0151] Song, X. , Y. Wang , and L. Gao . 2020. “Mechanism of Antioxidant Properties of Quercetin and Quercetin‐DNA Complex.” Journal of Molecular Modeling 26, no. 6: 133. 10.1007/s00894-020-04356-x. 32399900

[wrna70023-bib-0152] Sowndhararajan, K. , P. Deepa , M. Kim , S. J. Park , and S. Kim . 2018. “Neuroprotective and Cognitive Enhancement Potentials of Baicalin: A Review.” Brain Sciences 8, no. 6: 6. 10.3390/brainsci8060104. PMC6025220 29891783

[wrna70023-bib-0153] Sun, L. , X. Chen , S. Zhu , et al. 2024. “Hesperetin Derivative 2a Inhibits Lipopolysaccharide‐Induced Acute Liver Injury in Mice via Downregulation of circDcbld2.” Acta Pharmacologica Sinica 45, no. 2: 354–365. 10.1038/s41401-023-01171-x. 37845343 PMC10789727

[wrna70023-bib-0154] Sun, S. , and H. Fang . 2021. “Curcumin Inhibits Ovarian Cancer Progression by Regulating Circ‐PLEKHM3/miR‐320a/SMG1 Axis.” Journal of Ovarian Research 14, no. 1: 158. 10.1186/s13048-021-00916-8. 34784955 PMC8594156

[wrna70023-bib-0155] Sun, S.‐Q. , Y.‐X. Zhao , S.‐Y. Li , J.‐W. Qiang , and Y.‐Z. Ji . 2020. “Anti‐Tumor Effects of Astaxanthin by Inhibition of the Expression of STAT3 in Prostate Cancer.” Marine Drugs 18, no. 8: 8. 10.3390/md18080415. PMC7459748 32784629

[wrna70023-bib-0156] Szabo, L. , and J. Salzman . 2016. “Detecting Circular RNAs: Bioinformatic and Experimental Challenges.” Nature Reviews Genetics 17, no. 11: 679–692. 10.1038/nrg.2016.114. PMC5565156 27739534

[wrna70023-bib-0157] Tan, B. L. , M. E. Norhaizan , W.‐P.‐P. Liew , and H. Sulaiman Rahman . 2018. “Antioxidant and Oxidative Stress: A Mutual Interplay in Age‐Related Diseases.” Frontiers in Pharmacology 9: 1162. 10.3389/fphar.2018.01162. 30405405 PMC6204759

[wrna70023-bib-0158] Tao, X. , Y. Shao , J. Yan , et al. 2021. “Biological Roles and Potential Clinical Values of Circular RNAs in Gastrointestinal Malignancies.” Cancer Biology & Medicine 18, no. 2: 437–457. 10.20892/j.issn.2095-3941.2020.0348. 33710802 PMC8185857

[wrna70023-bib-0159] Tian, C. , X. Liu , Y. Chang , et al. 2021. “Investigation of the Anti‐Inflammatory and Antioxidant Activities of Luteolin, Kaempferol, Apigenin and Quercetin.” South African Journal of Botany 137: 257–264. 10.1016/j.sajb.2020.10.022.

[wrna70023-bib-0160] Vea, A. , V. Llorente‐Cortes , and D. De Gonzalo‐Calvo . 2018. “Circular RNAs: A Novel Tool in Cardiovascular Biomarker Development?” Non‐Coding RNA Investigation 2: 39. 10.21037/ncri.2018.06.06.

[wrna70023-bib-0161] Venkatas, J. , A. Daniels , and M. Singh . 2022. “The Potential of Curcumin‐Capped Nanoparticle Synthesis in Cancer Therapy: A Green Synthesis Approach.” Nanomaterials 12, no. 18: 18. 10.3390/nano12183201. PMC9502936 36144994

[wrna70023-bib-0162] Vollmannová, A. , T. Bojňanská , J. Musilová , J. Lidiková , and M. Cifrová . 2024. “Quercetin as One of the Most Abundant Represented Biological Valuable Plant Components With Remarkable Chemoprotective Effects—A Review.” Heliyon 10, no. 12: e33342. 10.1016/j.heliyon.2024.e33342. 39021910 PMC11253541

[wrna70023-bib-0163] Vono, R. , C. Fuoco , S. Testa , et al. 2016. “Activation of the Pro‐Oxidant PKCβII‐p66Shc Signaling Pathway Contributes to Pericyte Dysfunction in Skeletal Muscles of Patients With Diabetes With Critical Limb Ischemia.” Diabetes 65, no. 12: 3691–3704. 10.2337/db16-0248. 27600065

[wrna70023-bib-0164] Wang, J. , S. Liu , H. Wang , et al. 2019. “Xanthophyllomyces Dendrorhous‐Derived Astaxanthin Regulates Lipid Metabolism and Gut Microbiota in Obese Mice Induced by A High‐Fat Diet.” Marine Drugs 17, no. 6: 6. 10.3390/md17060337. PMC6627754 31195737

[wrna70023-bib-0165] Wang, M. , L. Sun , L. Wang , and Y. Sun . 2021. “Effects of Berberine on Circular RNA Expression Profiles in Human Gastric Cancer Cells.” Evidence‐Based Complementary and Alternative Medicine 2021: 1–16. 10.1155/2021/6688629. PMC8112944 34055022

[wrna70023-bib-0166] Wang, W. , Y. Xie , and A. Malhotra . 2021. “Potential of Curcumin and Quercetin in Modulation of Premature Mitochondrial Senescence and Related Changes During Lung Carcinogenesis.” Journal of Environmental Pathology, Toxicology and Oncology 40, no. 4: 53–60. 10.1615/JEnvironPatholToxicolOncol.2021039371. 34936300

[wrna70023-bib-0167] Wang, Y. , W. He , S. A. Ibrahim , Q. He , and J. Jin . 2021. “Circular RNAs: Novel Players in the Oxidative Stress‐Mediated Pathologies, Biomarkers, and Therapeutic Targets.” Oxidative Medicine and Cellular Longevity 2021: 6634601. 10.1155/2021/6634601. 34257814 PMC8245247

[wrna70023-bib-0168] Wang, Y.‐L. , L.‐L. Feng , J. Shi , et al. 2023. “CiRS‐7 Enhances the Liquid‐Liquid Phase Separation of miRISC and Promotes DNA Damage Repair.” Nucleus 14, no. 1: 2293599. 10.1080/19491034.2023.2293599. 38105528 PMC10730229

[wrna70023-bib-0169] Ward, A. B. , H. Mir , N. Kapur , D. N. Gales , P. P. Carriere , and S. Singh . 2018. “Quercetin Inhibits Prostate Cancer by Attenuating Cell Survival and Inhibiting Anti‐Apoptotic Pathways.” World Journal of Surgical Oncology 16, no. 1: 108. 10.1186/s12957-018-1400-z. 29898731 PMC6001031

[wrna70023-bib-0170] Wu, K. , Y. Wei , Y. Yu , M. Shan , Y. Tang , and Y. Sun . 2022. “Green Tea Polyphenols Inhibit Malignant Melanoma Progression via Regulating circ_MITF/miR‐30e‐3p/HDAC2 Axis.” Biotechnology and Applied Biochemistry 69, no. 2: 808–821. 10.1002/bab.2153. 33797132

[wrna70023-bib-0171] Wu, Y.‐L. , Z.‐J. Lin , C.‐C. Li , et al. 2023. “Epigenetic Regulation in Metabolic Diseases: Mechanisms and Advances in Clinical Study.” Signal Transduction and Targeted Therapy 8, no. 1: 1–27. 10.1038/s41392-023-01333-7. 36864020 PMC9981733

[wrna70023-bib-0172] Wu, Z. , M. Wu , X. Jiang , et al. 2024. “The Study on circRNA Profiling Uncovers the Regulatory Function of the hsa_circ_0059665/miR‐602 Pathway in Breast Cancer.” Scientific Reports 14, no. 1: 20555. 10.1038/s41598-024-71505-0. 39232183 PMC11374783

[wrna70023-bib-0173] Wu, Z. , X. Zhao , Y. Sun , and H. Yu . 2022. “Curcumin Suppresses Colorectal Cancer Development With Epithelial‐Mesenchymal Transition via Modulating Circular RNA HN1/miR‐302A‐3P/PIK3R3 Axis.” Journal of Physiology and Pharmacology 73: 219–231. 10.26402/jpp.2022.2.05. 35988930

[wrna70023-bib-0174] Xie, W. , M. Chu , G. Song , et al. 2022. “Emerging Roles of Long Noncoding RNAs in Chemoresistance of Pancreatic Cancer.” Seminars in Cancer Biology 83: 303–318. 10.1016/j.semcancer.2020.11.004. 33207266

[wrna70023-bib-0175] Xu, T. , J. Wu , P. Han , Z. Zhao , and X. Song . 2017. “Circular RNA Expression Profiles and Features in Human Tissues: A Study Using RNA‐Seq Data.” BMC Genomics 18, no. 6: 680. 10.1186/s12864-017-4029-3. 28984197 PMC5629547

[wrna70023-bib-0176] Xu, X. , J. Wang , X. Jin , et al. 2024. “Bu‐Shen‐Ning‐Xin Decoction Ameliorates Premature Ovarian Insufficiency by Suppressing Oxidative Stress Through rno_circRNA_012284/rno_miR‐760–3p/HBEGF Pathway.” Phytomedicine 133: 155920. 10.1016/j.phymed.2024.155920. 39126922

[wrna70023-bib-0177] Xu, X. , X. Zhang , Y. Zhang , and Z. Wang . 2021. “Curcumin Suppresses the Malignancy of Non‐Small Cell Lung Cancer by Modulating the Circ‐PRKCA/miR‐384/ITGB1 Pathway.” Biomedicine & Pharmacotherapy 138: 111439. 10.1016/j.biopha.2021.111439. 33684690

[wrna70023-bib-0178] Yaacoub, S. , A. Boudaka , A. AlKhatib , et al. 2024. “The Pharmaco‐Epigenetics of Hypertension: A Focus on microRNA.” Molecular and Cellular Biochemistry 479, no. 12: 3255–3271. 10.1007/s11010-024-04947-9. 38424404 PMC11511726

[wrna70023-bib-0179] Yang, B. , Y. Dong , F. Wang , and Y. Zhang . 2020. “Nanoformulations to Enhance the Bioavailability and Physiological Functions of Polyphenols.” Molecules 25, no. 20: 4613. 10.3390/molecules25204613. 33050462 PMC7587200

[wrna70023-bib-0180] Yang, P.‐W. , T.‐T. Chen , W.‐X. Zhao , et al. 2021. “Scutellaria Barbata D.Don and Oldenlandia Diffusa (Willd.) Roxb Crude Extracts Inhibit Hepatitis‐B‐Virus‐Associated Hepatocellular Carcinoma Growth Through Regulating circRNA Expression.” Journal of Ethnopharmacology 275: 114110. 10.1016/j.jep.2021.114110. 33864890

[wrna70023-bib-0181] Yao, C. , S. Dai , C. Wang , et al. 2023. “Luteolin as a Potential Hepatoprotective Drug: Molecular Mechanisms and Treatment Strategies.” Biomedicine & Pharmacotherapy 167: 115464. 10.1016/j.biopha.2023.115464. 37713990

[wrna70023-bib-0182] Yaribeygi, H. , S. L. Atkin , and A. Sahebkar . 2019. “Potential Roles of microRNAs in Redox State: An Update.” Journal of Cellular Biochemistry 120, no. 2: 1679–1684. 10.1002/jcb.27475. 30160790

[wrna70023-bib-0183] Yoshitomi, R. , M. Kumazoe , K.‐W. Lee , Y. Marugame , Y. Fujimura , and H. Tachibana . 2024. “Regulatory Effect of Epigallocatechin‐3‐O‐Gallate on Circular RNA Expression in Mouse Liver.” Journal of Nutritional Biochemistry 124: 109506. 10.1016/j.jnutbio.2023.109506. 37890708

[wrna70023-bib-0184] You, Z. , Y. Lei , Y. Yang , et al. 2025. “Therapeutic Target Genes and Regulatory Networks of Gallic Acid in Cervical Cancer.” Frontiers in Genetics 15: 1508869. 10.3389/fgene.2024.1508869. 39902297 PMC11789760

[wrna70023-bib-0185] Yu, G. , L. Chen , Y. Hu , Z. Yuan , Y. Luo , and Y. Xiong . 2021. “Antitumor Effects of Baicalein and Its Mechanism via TGFβ Pathway in Cervical Cancer HeLa Cells.” Evidence‐Based Complementary and Alternative Medicine 2021, no. 1: 5527190. 10.1155/2021/5527190. 33777154 PMC7979304

[wrna70023-bib-0186] Yu, X. , and Y. Liu . 2021. “Diosmetin Attenuate Experimental Ulcerative Colitis in Rats via Suppression of NF‐κB, TNF‐α and IL‐6 Signalling Pathways Correlated With Down‐Regulation of Apoptotic Events.” European Journal of Inflammation 19: 20587392211067292. 10.1177/20587392211067292.

[wrna70023-bib-0187] Yu, X. , Y. Liu , Y. Wang , X. Mao , Y. Zhang , and J. Xia . 2018. “Baicalein Induces Cervical Cancer Apoptosis Through the NF‐κB Signaling Pathway.” Molecular Medicine Reports 17, no. 4: 5088–5094. 10.3892/mmr.2018.8493. 29393414 PMC5865972

[wrna70023-bib-0188] Yu, Y. , Y. Xing , Q. Zhang , et al. 2021. “Soy Isoflavone Genistein Inhibits hsa_circ_0031250/ mir −873‐5p/ foxm1 Axis to Suppress Non‐Small‐Cell Lung Cancer Progression.” IUBMB Life 73, no. 1: 92–107. 10.1002/iub.2404. 33159503

[wrna70023-bib-0189] Yuan, L. , Y. Li , X. Li , et al. 2024. “The Molecular Mechanism of Naringin Improving Endometrial Receptivity of OHSS Rats.” Molecular Reproduction and Development 91, no. 8: e23715. 10.1002/mrd.23715. 37963204

[wrna70023-bib-0190] Yuan, Z. , J. Min , Y. Zhao , et al. 2018. “Quercetin Rescued TNF‐Alpha‐Induced Impairments in Bone Marrow‐Derived Mesenchymal Stem Cell Osteogenesis and Improved Osteoporosis in Rats.” American Journal of Translational Research 10, no. 12: 4313–4321. 30662673 PMC6325508

[wrna70023-bib-0191] Zaafan, M. A. , and A. M. Abdelhamid . 2021. “The Cardioprotective Effect of Astaxanthin Against Isoprenaline‐Induced Myocardial Injury in Rats: Involvement of TLR4/NF‐κB Signaling Pathway.” European Review for Medical and Pharmacological Sciences 25, no. 11: 4099–4105. 10.26355/eurrev_202106_26052. 34156689

[wrna70023-bib-0192] Zabady, S. , N. Mahran , M. A. Soltan , et al. 2022. “Cyanidin‐3‐Glucoside Modulates hsa_circ_0001345/miRNA106b/ATG16L1 Axis Expression as a Potential Protective Mechanism Against Hepatocellular Carcinoma.” Current Issues in Molecular Biology 44, no. 4: 1677–1687. 10.3390/cimb44040115. 35723373 PMC9164082

[wrna70023-bib-0193] Zeng, Y. , Y. Zou , G. Gao , et al. 2022. “The Biogenesis, Function and Clinical Significance of Circular RNAs in Breast Cancer.” Cancer Biology & Medicine 19, no. 1: 14–29. 10.20892/j.issn.2095-3941.2020.0485. PMC8763001 34110722

[wrna70023-bib-0194] Zhang, H. , H. Ye , H. Zhou , et al. 2024. “RNA‐Seq Analysis Revealed circRNAs Associated With Resveratrol‐Induced Apoptosis of Porcine Ovarian Granulosa Cells.” Cells 13, no. 18: 1571. 10.3390/cells13181571. 39329754 PMC11429535

[wrna70023-bib-0195] Zhang, J. , S. Chen , J. Yang , and F. Zhao . 2020. “Accurate Quantification of Circular RNAs Identifies Extensive Circular Isoform Switching Events.” Nature Communications 11: 90. 10.1038/s41467-019-13840-9. PMC6941955 31900416

[wrna70023-bib-0196] Zhang, K. , H. Zhuo , J. Guo , W. Wang , and R. Dai . 2024. “Astaxanthin Alleviates the Process of Cardiac Hypertrophy by Targeting the METTL3/Circ_0078450/MiR‐338‐3p/GATA4 Pathway.” International Heart Journal 65, no. 1: 119–127. 10.1536/ihj.23-423. 38296564

[wrna70023-bib-0197] Zhang, M. , H. Yang , E. Yang , J. Li , and L. Dong . 2021. “Berberine Decreases Intestinal GLUT2 Translocation and Reduces Intestinal Glucose Absorption in Mice.” International Journal of Molecular Sciences 23, no. 1: 327. 10.3390/ijms23010327. 35008753 PMC8745600

[wrna70023-bib-0198] Zhang, R. , Z. Hou , K. Liao , C. Yu , R. Jing , and C. Tu . 2022. “Expression Profile and Bioinformatics Analysis of Circular RNAs in Patients With Vitiligo.” Pharmacogenomics and Personalized Medicine 15: 785–796. 10.2147/PGPM.S371107. 36092681 PMC9451056

[wrna70023-bib-0199] Zhang, W. , Q. Liu , L. Luo , et al. 2021. “Use Chou's 5‐Steps Rule to Study How Baicalin Suppresses the Malignant Phenotypes and Induces the Apoptosis of Colorectal Cancer Cells.” Archives of Biochemistry and Biophysics 705: 108919. 10.1016/j.abb.2021.108919. 33992597

[wrna70023-bib-0200] Zhang, X. , S. Wang , H. Wang , et al. 2019. “Circular RNA circNRIP1 Acts as a microRNA‐149‐5p Sponge to Promote Gastric Cancer Progression via the AKT1/mTOR Pathway.” Molecular Cancer 18, no. 1: 20. 10.1186/s12943-018-0935-5. 30717751 PMC6360801

[wrna70023-bib-0201] Zhang, Y. , D. Chen , R. Tian , X. Yan , and Y. Zhou . 2023. “Resveratrol Alleviates Amyloid β‐Induced Neuronal Apoptosis, Inflammation, and Oxidative and Endoplasmic Reticulum Stress by circ_0050263/ mir −361‐3p/ pde4a Axis During Alzheimer's Disease.” Chemical Biology & Drug Design 102, no. 5: 1121–1132. 10.1111/cbdd.14313. 37620166

[wrna70023-bib-0202] Zhang, Y. , C. Yu , C. Peng , and F. Peng . 2024. “Potential Roles and Mechanisms of Curcumin and Its Derivatives in the Regulation of Ferroptosis.” International Journal of Biological Sciences 20, no. 12: 4838–4852. 10.7150/ijbs.90798. 39309443 PMC11414380

[wrna70023-bib-0203] Zhang, Z. , Q. Chen , C. Huang , et al. 2022. “Transcription Factor Nrf2 Binds to circRNAPIBF1 to Regulate SOD2 in Lung Adenocarcinoma Progression.” Molecular Carcinogenesis 61, no. 12: 1161–1176. 10.1002/mc.23468. 36193777

[wrna70023-bib-0204] Zhang, Z. , Y. Qiu , W. Li , et al. 2023. “Astaxanthin Alleviates Foam Cell Formation and Promotes Cholesterol Efflux in Ox‐LDL‐Induced RAW264.7 Cells via CircTPP2/miR‐3073b‐5p/ABCA1 Pathway.” Molecules 28, no. 4: 1701. 10.3390/molecules28041701. 36838686 PMC9961242

[wrna70023-bib-0205] Zhao, F. , L. Fu , W. Yang , et al. 2016. “Cardioprotective Effects of Baicalein on Heart Failure via Modulation of Ca2+ Handling Proteins In Vivo and In Vitro.” Life Sciences 145: 213–223. 10.1016/j.lfs.2015.12.036. 26706290

[wrna70023-bib-0206] Zhao, Y.‐H. , Z. Wang , N. Zhang , T. Cui , and Y.‐H. Zhang . 2020. “Effect of ciRS‐7 Expression on Clear Cell Renal Cell Carcinoma Progression.” Chinese Medical Journal 133, no. 17: 2084–2089. 10.1097/CM9.0000000000000867. 32496306 PMC7478654

[wrna70023-bib-0207] Zhen, H. , J. Shen , J. Wang , et al. 2022. “Characteristics and Expression of circ_003628 and Its Promoted Effect on Proliferation and Differentiation of Skeletal Muscle Satellite Cells in Goats.” Animals 12, no. 19: 2524. 10.3390/ani12192524. 36230263 PMC9559657

[wrna70023-bib-0208] Zheng, H. , X. Zhu , E. Gong , Y. Lv , Y. Li , and X. Cai . 2023. “Luteolin Suppresses Lung Cancer Progression Through Targeting the circ_0000190/miR‐130a‐3p/Notch‐1 Signaling Pathway.” Journal of Chemotherapy 35, no. 4: 330–342. 10.1080/1120009X.2022.2102303. 35943044

[wrna70023-bib-0209] Zheng, S. , X. Zhang , E. Odame , et al. 2021. “CircRNA—Protein Interactions in Muscle Development and Diseases.” International Journal of Molecular Sciences 22, no. 6: 3262. 10.3390/ijms22063262. 33806945 PMC8005172

[wrna70023-bib-0210] Zhou, Q. , G. Fang , Y. Pang , and X. Wang . 2023. “Combination of Kaempferol and Docetaxel Induces Autophagy in Prostate Cancer Cells In Vitro and In Vivo.” International Journal of Molecular Sciences 24, no. 19: 19. 10.3390/ijms241914519. PMC10572510 37833967

[wrna70023-bib-0211] Zhou, Y. , A. M. Richards , and P. Wang . 2019. “MicroRNA‐221 Is Cardioprotective and Anti‐Fibrotic in a Rat Model of Myocardial Infarction.” Molecular Therapy—Nucleic Acids 17: 185–197. 10.1016/j.omtn.2019.05.018. 31261033 PMC6606926

[wrna70023-bib-0212] Zhu, D. , M. Shao , J. Yang , et al. 2020. “Curcumin Enhances Radiosensitization of Nasopharyngeal Carcinoma via Mediating Regulation of Tumor Stem‐Like Cells by a CircRNA Network.” Journal of Cancer 11, no. 8: 2360–2370. 10.7150/jca.39511. 32127962 PMC7052922

[wrna70023-bib-0213] Zhu, W. , Y. Zhang , Q. Zhou , C. Zhen , H. Huang , and X. Liu . 2024. “Identification and Comprehensive Analysis of circRNA‐miRNA‐mRNA Regulatory Networks in A2780 Cells Treated With Resveratrol.” Genes 15, no. 7: 965. 10.3390/genes15070965. 39062744 PMC11276136

[wrna70023-bib-0214] Zinellu, A. , A. G. Fois , S. Sotgia , et al. 2016. “Plasma Protein Thiols: An Early Marker of Oxidative Stress in Asthma and Chronic Obstructive Pulmonary Disease.” European Journal of Clinical Investigation 46, no. 2: 181–188. 10.1111/eci.12582. 26681451

[wrna70023-bib-0215] Zuluaga, M. , V. Gueguen , D. Letourneur , and G. Pavon‐Djavid . 2018. “Astaxanthin‐Antioxidant Impact on Excessive Reactive Oxygen Species Generation Induced by Ischemia and Reperfusion Injury.” Chemico‐Biological Interactions 279: 145–158. 10.1016/j.cbi.2017.11.012. 29179950

